# ZFP36L1 promotes non‐small cell lung cancer progression under hypoxia by modulating CXCL9:SPP1 polarity: A single‐cell transcriptomic study

**DOI:** 10.1002/ctm2.70642

**Published:** 2026-03-29

**Authors:** Lijie Wang, Biao Chen, Jinxian He, Chengbin Lin, Weiyu Shen, Wang Lv, Luming Wang, Jian Hu

**Affiliations:** ^1^ Department of Thoracic Surgery, The First Affiliated Hospital Zhejiang University School of Medicine Hangzhou China; ^2^ Department of Thoracic Surgery The Affiliated Lihuili Hospital of Ningbo University Ningbo China

**Keywords:** CXCL9:SPP1, macrophage, non‐small cell lung cancer, single‐cell transcriptomics, ZFP36L1

## Abstract

**Background:**

Non‐small cell lung cancer (NSCLC) is the predominant lung cancer subtype with high mortality rate. Drug resistance and immune evasion limit its therapeutic outcomes. Specific mechanism of the oncogenic ZFP36L1 in NSCLC remains unclear.

**Methods:**

scRNA‐seq data were analysed using bioinformatic approaches. Hypoxia‐induced alterations in the macrophage CXCL9:SPP1 ratio were assessed by qRT‐PCR, Western blot (WB), immunofluorescence, flow cytometry and ELISA. Dual‐luciferase reporter and ChIP assays were used to validate ZFP36L1‐mediated transcriptional regulation of SPP1. In a co‐cultivation system of macrophages and NSCLC cells, tumour cell malignancy was evaluated through flow cytometry, CCK‐8, Transwell, colony formation and scratch assays. Patient‐derived organoids co‐cultured with macrophages were analysed via H&E, EdU and CellTiter‐Glo assays for pathological changes, proliferation and viability, as well as qRT‐PCR and WB for the expression of apoptosis‐related proteins. Finally, through a macrophage‐specific ZFP36L1‐knockout mouse model, the function of ZFP36L1 in CXCL9:SPP1 polarity and NSCLC progression was validated in vivo.

**Results:**

Hypoxia induced an imbalanced macrophage CXCL9:SPP1 ratio, with more pro‐tumour SPP1^+^ macrophages and fewer anti‐tumour CXCL9^+^ macrophages. Upregulation of ZFP36L1 promoted macrophage polarisation towards the SPP1^+^ phenotype, which then bound to CD44 on tumour cells to accelerate NSCLC progression.

**Conclusion:**

Under hypoxia, ZFP36L1 transcriptionally regulates SPP1 to reduce the macrophage CXCL9:SPP1 ratio, thereby driving NSCLC malignancy.

## Graphical abstract

1. Under hypoxia, downregulating the CXCL9:SPP1 ratio in macrophages promoted malignant progression of non‐small cell lung cancer (NSCLC).

2. Transcriptional factor ZFP36L1 modulated the CXCL9:SPP1 macrophage polarity.

3. ZFP36L1‐driven SPP1^+^ macrophages could accelerate NSCLC progression by binding to CD44 on the tumour cell surface.

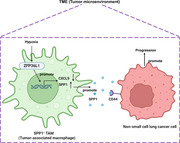

## 1 INTRODUCTION

Lung cancer constitutes a global health burden, causing nearly 2.5 million new cases and 1.8 million deaths per year. The incidence is substantially higher in men than in women.^1^ Non‐small cell lung cancer (NSCLC) comprises nearly 85% of all lung cancers.^2^ Chemotherapy, targeted therapy and immunotherapy are now applied in advanced NSCLC treatment.^3^, ^4^ Immune checkpoint blockage using EGFR, ALK and PD‐1/PD‐L1 inhibitors has improved prognosis substantially.^5^ However, drug resistance and tumour microenvironment (TME)‐mediated immune evasion continue to be major clinical challenges.^6^ Thus, identifying novel biomarkers and developing more effective therapeutic approaches are urgently needed.

The TME, composed of cancer‐associated fibroblasts, tumour cells, immune cells and endothelial cells (ECs), governs tumour initiation, progression and metastasis.^7^, ^8^ Tumour‐associated macrophages (TAMs) are predominant immune cells in the TME that contribute to inflammation, immune evasion and malignant progression.^9^ Recent scRNA‐seq studies have identified SPP1^+^ TAMs as a distinct macrophage subset linked with NSCLC progression.^10^, ^11^, ^12^ SPP1, a macrophage‐derived osteopontin, is involved in cell adhesion, migration and immune regulation.^13^ Reportedly, SPP1 mediates interactions between hepatocellular carcinoma (HCC) cells and macrophages via binding to CD44.^14^ The macrophage CXCL9:SPP1 ratio, can indicate either anti‐tumour or pro‐tumour TAM phenotypes.^15^ Regulators of CXCL9:SPP1 ratio in the TME, as well as the specific pathways, deserve further exploration.

ZFP36L1, a member of the ZFP36 gene family, promotes tumour progression in gastric cancer^16^ and glioma,^17^ and regulates immune responses in diverse immune cells.^18^ ZFP36L1 inhibits CD8^+^ T‐cell activation to modulate immune responses,^19^ and enhances IFN‐γ‐induced PD‐L1 expression via super‐enhancer‐mediated regulation.^20^ While it functions in multiple aspects of tumour and immune modulation, its role in macrophages and NSCLC malignancy has not been elucidated.

Through scRNA‐seq and cell experiments, our study demonstrates that hypoxia reduces the CXCL9:SPP1 ratio in macrophages and promotes NSCLC progression. ZFP36L1 is upregulated under hypoxia and serves as a key regulator of macrophage CXCL9:SPP1 ratio. Mechanistically, ZFP36L1‐induced SPP1^+^ macrophages enhance NSCLC progression via CD44 binding, providing a promising therapeutic target for NSCLC immunotherapy.

## 2 METHODS

### 2.1 Data collection

scRNA‐seq GSE148071 from the GEO (http://www.ncbi.nlm.nih.gov/geo/) was used, comprising 26 samples from patients with advanced NSCLC (two NSCLC, 11 LSCC and 13 LUAD). Additionally, four normal lung tissue clinical samples from GSE131907 served as controls.

The RNA sequencing data (TPM) of gene expression and clinical information for TCGA_LUAD were from UCSC Xena (https://gdc.xenahubs.net). Gene expression files, together with the corresponding gene symbol relationships, were converted into Gene ID. In TCGA_LUAD dataset, we grouped the samples using the optimal cutoff. The optimal cutoff for samples sorted into better and worse survival groups was determined by ‘surv_cutpoint’ in the ‘survminer’ R package. Overall survival (OS) and PFI of the samples underwent survival curve analysis.

Gene expression matrix for GSE3141 was downloaded from the GEO, with probes converted into Gene Symbols. In the GSE3141 dataset, we categorised the samples based on the optimal cutoff and conducted survival curve analysis on their OS.

### 2.2 Single‐cell data processing and quality control

Data from 30 samples were integrated, yielding an initial dataset of 83,417 cells and 18,197 genes. For subsequent analyses, the ‘Seurat’ R package (v4.1.0) was applied. Following quality control (QC) was applied: (1) unique gene counts and unique molecular identifiers per cell were required to be >100, with no upper limit; (2) mitochondrial genes per cell were required to be <15%; and (3) cells with nCount >50 000 or nFeature >7000 were excluded. The final dataset consisted of 80 750 cells and 18 197 genes.

### 2.3 Cell clustering and annotation

The scaled expression matrix was subjected to PCA with the ‘RunPCA’ function. Cell clusters were constructed from the first 40 principal components (PCs) with ‘FindNeighbors’ and ‘FindClusters’ functions, visualised by UMAP. Cell annotations and subtyping referenced canonical gene markers from the published literature^21^ and the CellMarker 2.0 database. The distribution of marker genes was analysed.

### 2.4 Transcription factor analysis

The KnockTF 2.0 database was employed to identify transcription factors (TFs) that regulate SPP1 and CXCL9TFs regulating SPP1 and CXCL9. Marker genes for SPP1^+^ and CXCL9^+^ TAMs were obtained by the ‘FindMarkers’ function in Seurat for the intersection with the TFs. The expression of key genes among SPP1^+^, CXCL9^+^ and SPP1^−^/CXCL9^−^ TAMs was assessed through correlation analysis.

### 2.5 Pathway enrichment analysis

Standard gene sets were all from the MSigDB. Pathway activity was evaluated with the ‘GSVA’ R package. Mean gene expression in each cluster was quantified using the ‘Seurat’ package. Significant enrichment was defined by an Adj. p.val <.05. Key pathways were visualised with the ‘ggplot2’ package.

### 2.6 Cell–cell communication

Seurat objects were normalised and imported into the CellChat toolkit. The ‘computeCommunProb’ function was used to calculate communication probabilities. Significant ligand–receptor interactions within given signalling pathways and signal transduction genes were identified using the ‘extractEnrichedLR’ function to reveal communication numbers and strength among cell populations. The overall communication patterns and signalling networks were visualised and analysed using built‐in CellChat functions.

### 2.7 Cell cultivation

Human monocytic leukaemia THP‐1 cells (CL‐0233, Procell), human NSCLC A549 cells (BNCC337696), human LSCC NCI‐H2170 cells (BNCC101664) and mouse Lewis lung carcinoma (LLC) cells (BNCC100069, all from BeNa Culture Collection) were used. THP‐1 cells were cultivated in RPMI‐1640 with 10% foetal bovine serum (FBS) (Gibco) and  .05 mM β‐mercaptoethanol (Maokangbio), NCI‐H2170 cells in 20% FBS‐containing RPMI‐1640, A549 cells in 10% FBS‐containing F‐12K medium and LLC cells in 10% FBS‐containing DMEM‐H. All media were from Gibco and contained 1% P‐S (Beyotime). The condition for cell incubation was 37°C and 5% CO_2_.

THP‐1 cells were differentiated to M0 macrophages with 150 nM PMA (Sigma–Aldrich) for 48 h. Hypoxia was mimicked as 1% O_2_, 5% CO_2_ and 94% N_2_ using a Tri‐gas incubator (Thermo Fisher). After the macrophages were treated under hypoxic conditions for 48 h, the following functional experiments and co‐culture experiments were conducted. The supernatant from the resulting M0 macrophages was co‐cultivated with tumour cells at a ratio of 1:5 for 48 h.

### 2.8 Cell transfection

Short hairpin RNAs targeting ZFP36L1 (sh‐ZFP36L1 or sh‐ZFP36L1#1) and SPP1 (sh‐SPP1), ZFP36L1 overexpression plasmid (oe‐ZFP36L1), and their controls (sh‐NC and oe‐NC) were synthesised by GenePharma. Transfections into THP‐1 cells continued with Lipofectamine 3000 (Invitrogen) for 48 h before use.

### 2.9 qRT‐PCR

Total RNA extracted with TRIzol reagent (Invitrogen) were synthesised into cDNA with the HiScript Q RT SuperMix kit (Vazyme), which were amplified on an ABI 7500 system with SYBR Green Premix (MCE) (primers in Table 1). Gene expression was computed by the 2^−ΔΔCt^, normalised to β‐actin.

**TABLE 1:** Primer sequences for qRT‐PCR.

Gene	Forward primer (5′–3′)	Reverse primer (5′–3′)
SPP1	AGCTTTACAACAAATACCCAGATGC	GGACTTACTTGGAAGGGTCTGTG
CXCL9	ATTGGTGCCCAGTTAGCCTC	TTCTGGCCACAGACAACCTC
Caspase 3	ACTGGACTGTGGCATTGAGA	GCACAAAGCGACTGGATGAA
Bcl‐2	AAAAATACAACATCACAGAGGAAGT	GTTTCCCCCTTGGCATGAGA
ZFP36L1	GCATGGCTGATTCAACTCCA	CCTCGCCTTTCTGACTCCG
β‐Actin	CAAAGTTCACAATGTGGCCG	TGGCAAGGGACTTCCTGTAAC

### 2.10 Western blot

Extracted using RIPA lysis buffer (Beyotime), total protein was denatured at 100°C for 5 min in loading buffer (Beyotime), separated by SDS‐PAGE, and moved to PVDF membranes (Sigma–Aldrich). After 5% non‐fat milk blocking (45 min), membranes were kept with primary antibodies at 4°C overnight; washed three times with TBST, HRP‐conjugated secondary antibodies were added (1 h, room temperature [RT]). With a Tanon‐4600 imaging system (Biotanon), protein bands were visualised. Antibody information is detailed in Table 2.

**TABLE 2:** Antibodies for Western blot.

Antibody	Manufacturer	Cat. no.
Anti‐CXCL9 (rabbit)	Abcam (UK)	ab290643
Anti‐SPP1 (mouse)	Invitrogen (USA)	MA5‐17180
Anti‐ZFP36L1 (rabbit)	Abcam (UK)	ab230507
Anti‐MMP9 (rabbit)	Abcam (UK)	ab76003
Anti‐MMP12 (rabbit)	Invitrogen (USA)	MA5‐24851
Anti‐Caspase 3 (rabbit)	Abcam (UK)	ab32351
Anti‐Bcl‐2 (rabbit)	Abcam (UK)	ab182858
Anti‐β‐actin (rabbit)	Abcam (UK)	ab213262
Goat anti‐mouse IgG H&L (HRP)	Abcam (UK)	ab205719
Goat anti‐rabbit IgG H&L (HRP)	Abcam (UK)	ab6721

### 2.11 Immunofluorescence

For M0 macrophages, cells on sterile coverslips in 24‐well plates were subjected to three PBS washings, 75% ethanol fixation (15 min) and  .1% Triton X‐100 (Beyotime) permeabilisation (15 min). After 1 h of 3% BSA (Beyotime) blocking, incubation with primary antibodies continued at 4°C overnight; after PBS washings, at RT without lights, fluorophore‐conjugated secondary antibodies were introduced for 1 h. After counterstaining with DAPI (Beyotime), nuclei were imaged with a fluorescence microscope (Olympus).

For mouse tissues, sections were immobilised in 4% paraformaldehyde (PFA, Beyotime) for 24 h, dehydrated, embedded and sectioned into 5 µm. After 1 h of 3% BSA blocking 1 h at RT, primary antibodies were applied (4°C, overnight), and after three TBST washes, secondary antibodies for 1 h at RT. After DAPI (Beyotime) counterstaining, under a fluorescence microscope tissues were recorded (Olympus). Antibodies for immunofluorescence (IF) are detailed in Table 3.

**TABLE 3:** Antibodies for immunofluorescence.

Antibody	Manufacturer	Cat. no.
Anti‐CXCL9 (rabbit)	Invitrogen (USA)	701117
Anti‐SPP1 (mouse)	Invitrogen (USA)	MA5‐17180
Anti‐SPP1 (rabbit)	Proteintech (China)	22952‐1‐AP
Anti‐F4/80 (mouse)	Santa Cruz Biotechnology (USA)	sc‐377009
Goat anti‐mouse IgG H&L (Alexa Fluor 488)	Abcam (UK)	ab150113
Goat anti‐rabbit IgG H&L (Alexa Fluor 555)	Abcam (UK)	ab150078

### 2.12 Flow cytometry

Apoptosis detection used a flow cytometer (BD Biosciences). Cells digested with trypsin without EDTA (Beyotime) were centrifuged and rinsed in PBS twice, before being resuspended in binding buffer (500 µL) and stained with Annexin V‐FITC and PI (each for 5 µL, 10 min, RT; provided by kit HY‐K1073, MCE).

For the detection of SPP1^+^/CXCL9^+^ TAMs, cells were immobilised with 4% PFA (Beyotime) for 1 h at RT. After two washes with pre‐cooled permeabilisation buffer, cells were kept with anti‐CXCL9 (ab290643) and goat anti‐rabbit IgG Fc (Alexa Fluor 750) (ab175735, both from Abcam) or anti‐SPP1 (6696‐RBM3‐P0, Thermo Fisher) for 1 h at RT without lights. Permeabilisation buffer washing of cells continued twice. Resuspended cells in buffer were assessed on a flow cytometer (BD Biosciences).

### 2.13 ELISA

IL‐7, CXCL10, IL‐1β and CXCL5 levels were tested with commercial ELISA kits. Cell supernatants or serum samples were centrifuged (10 min, 2000 rpm, 4°C) before resulting supernatants stored on ice. Then, 100 µL of standards or samples was incubated in a 96‐well plate for 2 h at 37°C before HRP‐conjugated antibody (100 µL/well) being applied for 1 h at 37°C. After rising with 200 µL wash buffer, wells stood for 2 min. Colours were developed with 90 µL of TMB substrate for 30 min without lights. Reaction was stopped by 50 µL of stop solution before measuring optical density (OD; 450 nm) on a microplate reader (Bio‐Rad). CXCL10 (mouse: E‐EL‐M0021; human: E‐EL‐H0050), IL‐1β (mouse: E‐EL‐M0037; human: E‐EL‐H0149) and CXCL5 (mouse: E‐EL‐M0471; human: E‐EL‐H0046) ELISA kits from Elabscience (China) and IL‐7 ELISA kits (mouse: EMIL7; human: EHIL7) from Thermo Fisher were used.

### 2.14 CCK‐8 viability assay

Single‐cell suspension was prepared with logarithmic phase co‐cultured cells trypsinised (Beyotime) at 2000 cells/mL (100 µL) and incubated in a 96‐well plate, for 0, 24, 48, 72 and 96 h, before reaction with CCK‐8 reagent (10 µL/well; provided by kit, Beyotime) for 1–4 h. OD (450 nm) was tested with a microplate reader (Bio‐Rad).

### 2.15 Colony formation assay

Log‐phase cells (100 cells/well) were put into 12‐well plates in 1 mL of culture medium. Every 3 days, medium was replaced with a new one and after 14 days was removed. After two gentle PBS washes, colonies were immobilised with 4% PFA for 20 min at RT, stained with  .1% crystal violet solution (15 min; Beyotime). After removing excess dye with three PBS rinses, the plates were air‐dried at RT. Colonies were imaged with ImageJ (NIH).

### 2.16 Scratch assay

At an appropriate density, cells were put into six‐well plates. Upon approximately 95% confluence, a sterile 10 µL pipette tip was used to create a straight scratch in the monolayer. Detached cells were removed by three PBS washes. Scratch was imaged at 0 and 48 h using an inverted microscope (Olympus) and quantified for closure rate by ImageJ (NIH).

### 2.17 Transwell assays

Cell migration and invasion abilities were tested 24 h post‐transfection. After trypsin (Beyotime) digestion, cells were resuspended in serum‐free medium and adjusted to 1 × 10^4^ cells/mL. To detect migration, the upper and lower chambers of the Transwell system (Corning) were filled with cell suspension (200 µL) and complete medium (500 µL) containing 10% FBS, respectively. To detect invasion, Transwell inserts were pre‐coated with Matrigel (BD Biosciences). The incubation lasted for 24 h at 37°C. With a wet cotton swab, we wiped off non‐migrated/invaded cells. Cells on the lower surface were immobilised with 4% PFA (Beyotime) for 20 min, followed by 15 min of  .5% crystal violet (Beyotime) staining. After PBS rinse, cells on the lower membrane surface were recorded with an inverted microscope (Olympus).

### 2.18 ChIP assay

Cells were collected and cross‐linked with 1% formaldehyde for 10 min. The cross‐linking was quenched by 5 min of glycine treatment (MCE; final concentration:  .125 M) at RT. The cross‐linked chromatin was then fragmented by sonication (300–500 bp). Immunoprecipitation was performed by incubating the sonicated samples with Protein A/G magnetic beads (HY‐K0202, MCE) pre‐adsorbed by specific rabbit anti‐ZFP36L1 (ZFP36L1‐101AP, Invitrogen) or IgG isotype control (ab313801, Abcam). Target DNA was finally amplified and detected by qPCR.

### 2.19 Dual‐luciferase reporter assay

ZFP36L1 regulation of the SPP1 promoter was analysed. Wild‐type (WT) and mutant (MUT) promoter fragments of SPP1 were separately cloned into the pGL4Luc‐RLuc dual‐luciferase vector (Novopro). One microgram of reporter plasmid was co‐transfected with oe‐ZFP36L1 or oe‐NC into 293T cells by Lipofectamine 3000 (Invitrogen). Firefly and Renilla luciferase activities were quantified 48 h later with a commercial kit (Promega) and expressed as Firefly/Renilla ratio.

### 2.20 Detection of mRNA stability

The processed macrophages were inoculated in six‐well plates and 5 µg/mL of actinomycin D was added. The cells were treated for 0, 2, 4 and 8 h, respectively. Cells were collected for qRT‐PCR.

### 2.21 Patient‐derived organoids

Under the approval conferred by the Ethics Committee of Ningbo Medical Center Lihuili Hospital (approval number KY2025SL501‐01), we gathered tumour and adjacent normal tissues from 40 NSCLC patients. Informed consent of patients was obtained.

Inclusion criteria: age ≥18 years; histopathologically confirmed NSCLC with tissue specimens obtained via fiberoptic bronchoscopy biopsy, pretreatment lung puncture or surgical resection; clinical stage IIIB–IV or recurrent/metastatic disease; absence of severe comorbidities; at least one measurable lesion according to response evaluation criteria in solid tumours; and an Eastern Cooperative Oncology Group scoring of 0–2.

Exclusion criteria: presence of other primary tumours outside the lung; previous adoptive cell therapy, tumour vaccines or other forms of immunotherapy; previous systemic immunosuppressive therapy; or a history of non‐infectious pneumonia or active autoimmune disease requiring steroid treatment.

### 2.22 Patient‐derived organoid cell culture

Fresh NSCLC samples were minced into fragments (≤1 mm^3^) and rinsed three times with pre‐cooled PBS containing 10% P‐S (Beyotime). The tissue fragments were digested in pre‐cooled digestion buffer, which contains 1% FBS (Gibco), 1.5 mg/mL collagenase type II (Thermo Fisher), 500 U/mL collagenase type IV (Thermo Fisher),  .1 mg/mL Dispase II (Beyotime) and 10 µM Y‐27632 (MCE) at 37°C with agitation for 45 min. The digest was centrifuged at 200 × *g* for 5 min. Three PBS washings were run on the pellet. The final single‐cell suspension was mixed with Matrigel (BD Biosciences) and seeded into 96‐well plates.

Upon Matrigel polymerisation, organoids were switched to modified organoid complete medium, that is, advanced DMEM/F12 (Gibco) containing N‐acetyl‐L‐cysteine (1 mM, Yeasen), gastrin (10 nM, Sigma–Aldrich), Noggin (.1 µg/mL), R‐spondin (1 µg/mL), EGF (50 ng/mL), nicotinamide (10 mM), A83‐01 (.5 µM) and FGF10 (100 ng/mL; MCE), and standard supplements (GlutaMAX, HEPES, N2, B27; Thermo Fisher). Media were refreshed every 3 days; organoids were passaged every 7 days according to growth.

### 2.23 Organoid‐macrophage co‐culture

Pre‐thawed Matrigel was immobilised with pre‐cooled basal medium at 2:3. Using a Transwell system (Corning), 50 µL of the mixture was spread over the upper inserts and incubated at 37°C for 1 h. After the Matrigel had fully solidified, the organoid cell suspension was seeded onto the inserts. The Transwell system was placed into a 24‐well plate pre‐seeded with M0 macrophages. They were co‐cultured in an appropriate amount of complete medium in a 37°C, 5% CO_2_ incubator for 48 h. Upon the completion of co‐culture, Transwell inserts were carefully taken out and organoid cells were collected for use.

### 2.24 H&E staining

Tissues were immobilised by 4% PFA (Beyotime) for 24 h, paraffin‐embedded conventionally and sliced into serial 5 µm sections with a microtome. After deparaffinisation and rehydration, haematoxylin (Beyotime) staining of the section lasted for 5–10 min. The sections were differentiated with 1% acid alcohol for 1–3 s, rinsed in tap water for several seconds to remove excess stain, blued in 1% ammonia water for 10 s, and rinsed in tap water for about 10 min. The sections were counterstained with eosin (Beyotime) for 1–2 min. After graded ethanol dehydration, xylene clearing and neutral balsam mounting, sections were observed with an optical microscope (Olympus).

### 2.25 EdU staining

The organoid cell suspension was seeded into 24‐well plates containing circular coverslips and cultured overnight at 37°C to allow attachment. Then, 1× EdU working solution (CA1170, Solarbio) was put into each well and incubated for 2 h at 37°C. After discarding the media, 4% PFA (Beyotime) was applied to fix cells for 15 min at RT, followed by 10 min of  .3% Triton X‐100 (Beyotime) permeabilisation. After three PBS washes, 100 µL of click reaction mixture (Beyotime) was introduced for 30 min RT incubation, protected from the light. After another wash, DAPI (Beyotime) was added and incubated for 10 min without lights. Finally, images were captured with a fluorescence microscope (Olympus).

### 2.26 CellTiter‐Glo assay

Organoid viability was tested with the CellTiter‐Glo assay kit (G7570, Promega). Briefly, 5000 organoid cells were put into 96‐well plates. The cultivation lasted for 0, 24, 48, 72 and 96 h. Each well received 80 µL of CellTiter‐Glo detection reagent. The plate was shaken at RT for 2 min without lights to mix and then stood for 10 min. Luminescence intensity was tested by a multifunctional microplate reader (Bio‐Rad).

### 2.27 Laboratory animals

C57BL/6J mice with macrophage‐specific conditional knockout of ZFP36L1 were produced by GemPharmatech. The study involved crossing LysM‐Cre mice with Zfp36l1^fl/fl^ mice to obtain macrophage/myeloid cell‐specific ZFP36L1 knockout mice (LysM‐Cre±; Zfp36l1^fl/fl^, hereinafter referred to as ZFP36L1‐KO). The control group consisted of littermate LysM‐Cre–/–; Zfp36l1^fl/fl^ WT mice (hereinafter referred to as WT). Mice (total number = 12) were housed under SPF conditions at the Ningbo Medical Center Lihuili Hospital Laboratory Animal Center, maintained at 22 ± 2°C and 50 ± 10% humidity with a 12/12‐h light/dark cycle. Mice were subcutaneously injected with 1 × 10^6^ of LLC cells (*n* = 6 per group). Tumour growth was monitored on days 7, 14, 21 and 28. Tumour length (*L*) and width (*W*) were tested with calipers to calculate tumour volume (*V*): *V* = *L* × *W* ^2^/2. After 4 weeks, mice were euthanased to dissect tumours and record their volume and weight. The animal protocol was completed under the approval conferred by the Ethics Committee of Zhejiang Luoxi Medical Technology Co., Ltd., Hangzhou, China (approval number LX4825021301).

### 2.28 Macrophage isolation

Bone marrow cells were utilised and red blood cells were removed with red blood cell lysis buffer (Beyotime). To assess cell purity, flow cytometry was conducted, with mouse anti‐F4/80 (11‐4801‐82, Invitrogen) used. Bone marrow‐derived macrophages with >85% F4/80^+^ purity were used for subsequent experiments.

### 2.29 Immunohistochemistry

Clinical tissue samples and fresh tumour tissues from mice were collected, immobilised with 10% formalin (Maokangbio) for 24 h, respectively, paraffin‐embedded and sectioned. Deparaffinised and rehydrated sections underwent antigen retrieval in  .01 M sodium citrate buffer (pH 6.0; Meilunbio). After blocking endogenous peroxidase with 3% H_2_O_2_ (Maokangbio) and nonspecific sites with 5% BSA (MCE), sections were kept with primary antibodies (4°C, overnight) and HRP‐conjugated secondary antibodies (1 h) after three PBS washes. Signals were tested with a DAB substrate kit (Sigma–Aldrich). Nuclei were counterstained with haematoxylin (Beyotime). After dehydration with ethanol and clearing with xylene, sections were mounted with neutral balsam for photos under a microscope (Olympus). Antibodies for immunohistochemistry (IHC) are detailed in Table 4.

**TABLE 4:** Antibodies for immunohistochemistry.

Antibody	Manufacturer	Cat. no.
Anti‐ZFP36L1 (rabbit)	Cusabio (China)	CSB‐PA004298
Anti‐CXCL9 (rabbit)	Abcam (UK)	ab320827
Anti‐SPP1 (rabbit)	Abcam (UK)	ab218237
Anti‐CD44 (rabbit)	Abcam (UK)	ab243894
Anti‐Ki67 (rabbit)	Abcam (UK)	ab16667
Goat anti‐rabbit IgG H&L (HRP)	Abcam (UK)	ab6721

### 2.30 Statistical analysis

Data (mean ± SD) were obtained from independent experiments (minimal count: 3). Statistical analyses and visualisation were run in GraphPad Prism 8.0 (GraphPad Software). Between‐group difference was tested by Student's *t*‐test or one‐way ANOVA (*p*‐value < .05: statistical significance).

## 3 RESULTS

### 3.1 Analysis of macrophage CXCL9:SPP1 ratio in NSCLC based on single‐cell datasets

**FIGURE 1:**
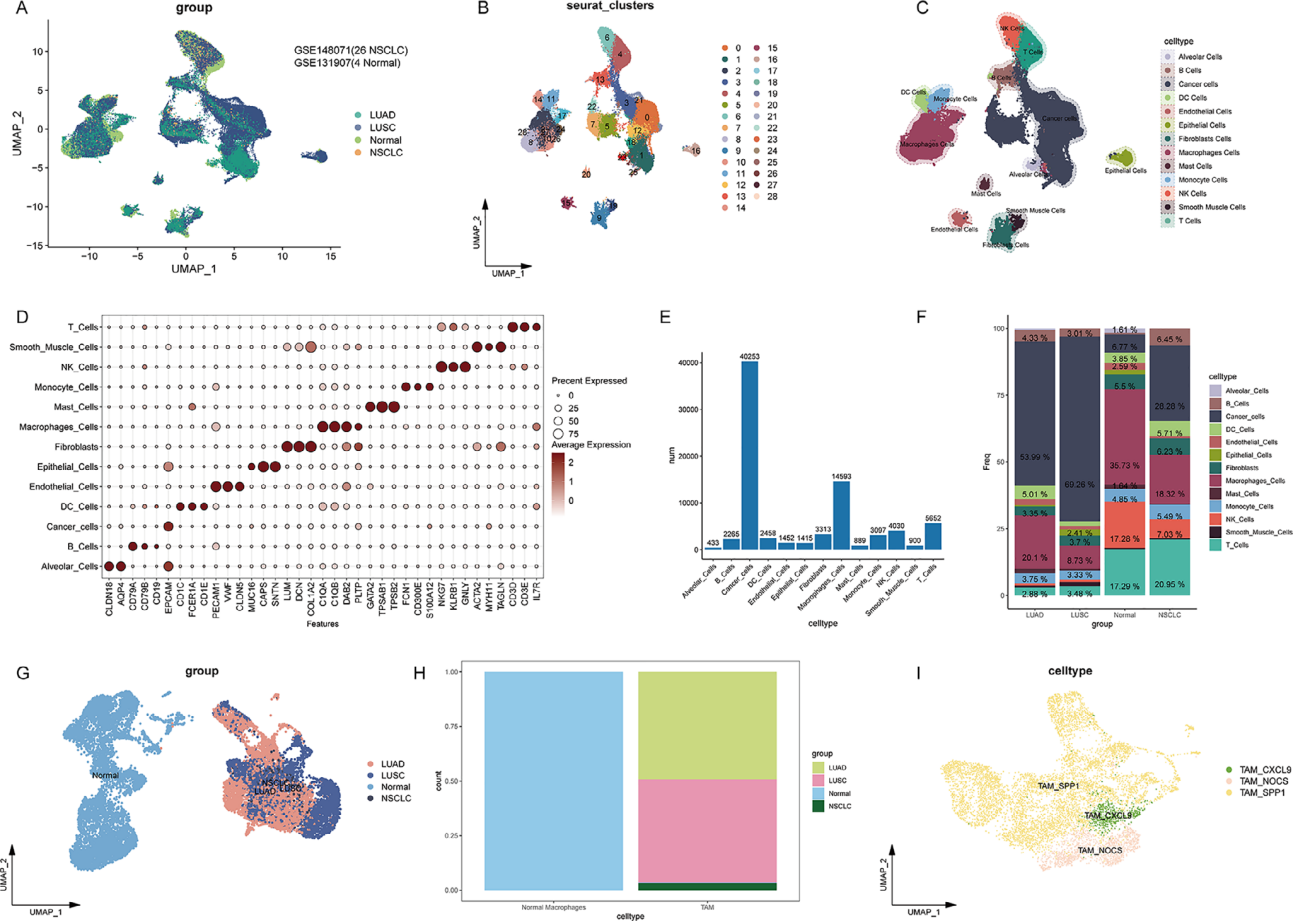
Cell clustering and annotation of non‐small cell lung cancer (NSCLC) cells based on scRNA‐seq. (A) Source and types of single cell quantities. (B) UMAP clustering results of all cells. (C) Annotation results of all cells. (D) Marker gene expression for each cell cluster. (E) Analysis of cell quantities. (F) Analysis of cell proportions. (G) Classification UMAP of macrophage subpopulations. (H) Proportions of macrophage subpopulations. (I) Annotation results of tumour‐associated macrophage (TAM) cells.

We first analysed 26 advanced NSCLC samples from the GSE148071 dataset and four normal tissue samples from the GSE131907 dataset. QC for the raw scRNA‐seq data (nFeature, nCount and mitochondrial gene percentage) is displayed in violin plots (Figure S1A). Point density plots illustrate the correlation between gene expression features and mitochondrial content before filtering (Figure S1B). Gene expression characteristics of retained cells after QC are shown in Figure S1C,D. Following data normalisation, 3000 highly variable genes were identified (Figure S1E). PCA effectively reduced data dimensionality and identified key PCs (Figure S1F). Using the top 40 PCs, cells were partitioned into 28 clusters through the ‘FindNeighbors’ and ‘FindClusters’ functions. The proportion distribution of each cell type and the characteristics of the sample sources are shown in Figure 1A,B. Based on marker genes from published literature^21^ and the CellMarker 2.0 database, we annotated 13 major cell types: T cells, ECs, monocytes, dendritic cells, B cells, mast cells, smooth muscle cells, NK cells, macrophages, epithelial cells, alveolar cells, fibroblasts and tumour cells (Figure 1C,D). We also visualised the proportions of each cell type (Figure 1E,F). After extracting the above macrophages and conducting re‐dimensionality reduction and clustering analysis, two subgroups were finally identified: normal macrophages and TAM (Figures 1G and S1G–I). The proportions of normal macrophages and TAM in distinctive samples are shown in Figure 1H. Then, the TAM subgroups were further clustered, and three subgroups were identified: SPP1^+^ TAM (high expression of SPP1), CXCL9^+^ TAM (high expression of CXCL9) and CPM^+^ TAM (SPP1/CXCL9 double‐negative), with SPP1^+^ TAM accounting for the majority (Figure 1I). These findings indicate that TAM demonstrates significant heterogeneity in the NSCLC microenvironment.

### 3.2 Hypoxia decreases the macrophage CXCL9:SPP1 ratio and promotes NSCLC progression

**FIGURE 2:**
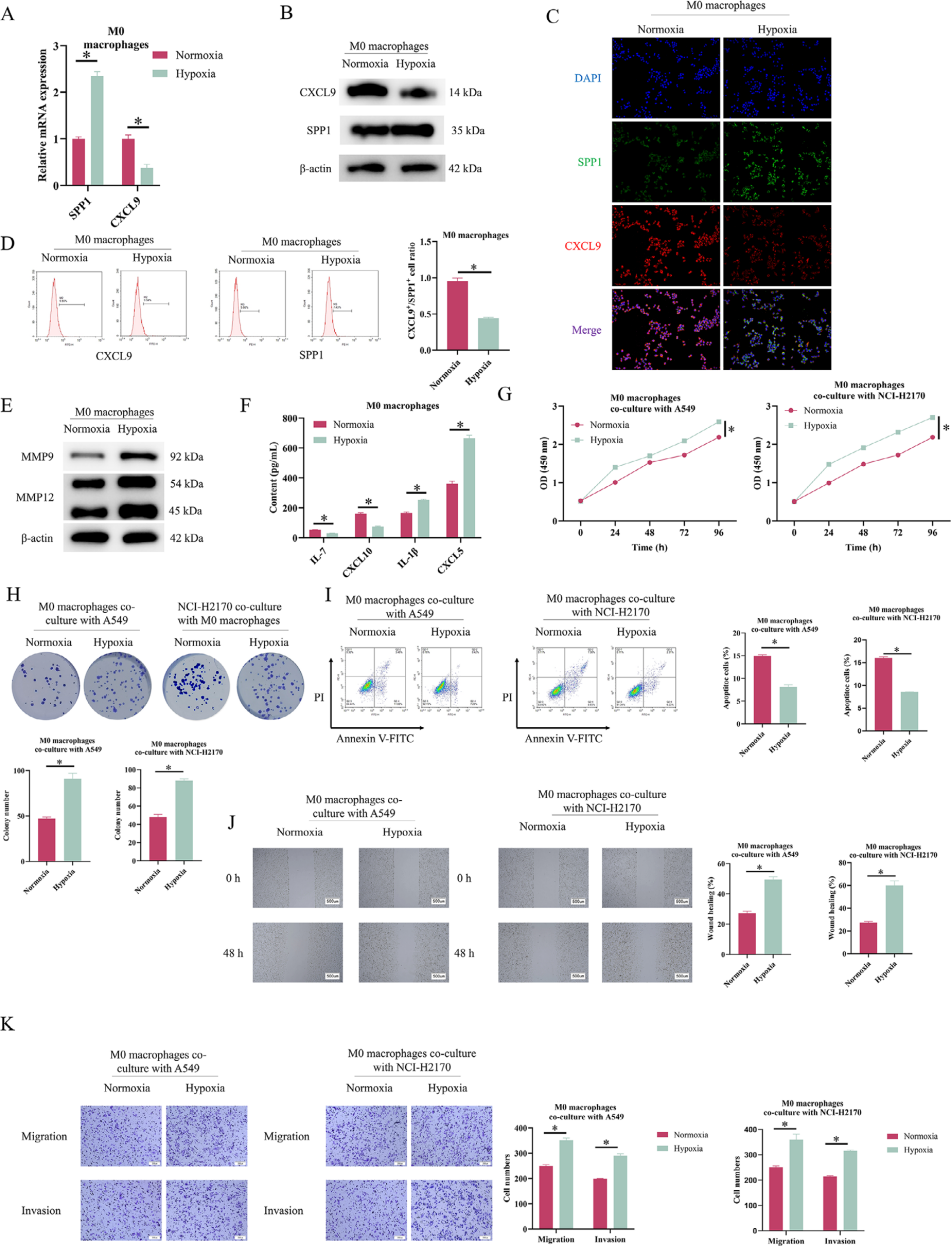
Hypoxia decreases the macrophage CXCL9:SPP1 ratio and promotes non‐small cell lung cancer (NSCLC) progression. M0 macrophages induced by PMA using THP‐1 cells were cultured under normoxia or hypoxia. (A) qRT‐PCR and (B) Western blotting (WB) of SPP1 and CXCL9 mRNA and protein levels. (C) Immunofluorescence staining assessing CXCL9 and SPP1 expression. (D) Flow cytometric analysis of CXCL9^+^/SPP1^+^ macrophage ratio. (E) WB analysis of MMP9 and MMP12 protein levels. (F) ELISA of IL‐7, CXCL10, IL‐1β and CXCL5 levels. M0 macrophages under normoxia or hypoxia were separately co‐cultured with A549 or NCI‐H2170 cells. (G) CCK‐8 viability assay. (H) Colony formation assay for cell proliferation. (I) Flow cytometric analysis of apoptosis. (J) Scratch migration assay. (K) Transwell migration and invasion assays. ^*^ *p* < .05.

The generation of SPP1^+^ TAMs is closely associated with a hypoxic microenvironment.^15^ The impact of macrophage CXCL9:SPP1 ratio on NSCLC progression was investigated. First, M0 macrophages were differentiated from THP‐1 cells using PMA and cultivated in either normoxia or hypoxia. qRT‐PCR and Western blot (WB) revealed that hypoxia suppressed CXCL9 expression while increasing both SPP1 mRNA and protein levels (Figure 2A,B), which was confirmed by IF staining (Figure 2C). Flow cytometry revealed a decreased proportion of CXCL9^+^/SPP1^+^ macrophages in the hypoxic group (Figure 2D), confirming that hypoxia induced a decrease in the CXCL9:SPP1 ratio. MMP9 and MMP12 are reported as markers of SPP1^+^ TAMs.^22^ WB results demonstrated that hypoxia significantly enhanced MMP9 and MMP12 protein expression (Figure 2E), which is in line with the induction of SPP1^+^ TAMs. CXCL9, CXCL10 and IL‐7 are enriched in the high CXCL9:SPP1 ratio subset, whereas CXCL5 and IL‐1β dominate the low‐ratio population.^15^ In hypoxic macrophages, ELISA revealed decreased production of IL‐7 and CXCL10 while increasing IL‐1β and CXCL5 levels (Figure 2F). To probe into the effect of hypoxia‐induced macrophage polarisation on tumour cell behavior, we co‐cultivated normoxia/hypoxia‐treated macrophages with A549 or NCI‐H2170 cells independently. In functional assays, hypoxic macrophages enhanced tumour cell proliferation, migration and invasion, while inhibiting apoptosis (Figure 2G–K). In conclusion, hypoxia promotes NSCLC malignant progression by reducing the macrophage CXCL9:SPP1 ratio.

### 3.3 ZFP36L1 is a key gene regulating the macrophage CXCL9:SPP1 ratio

**FIGURE 3:**
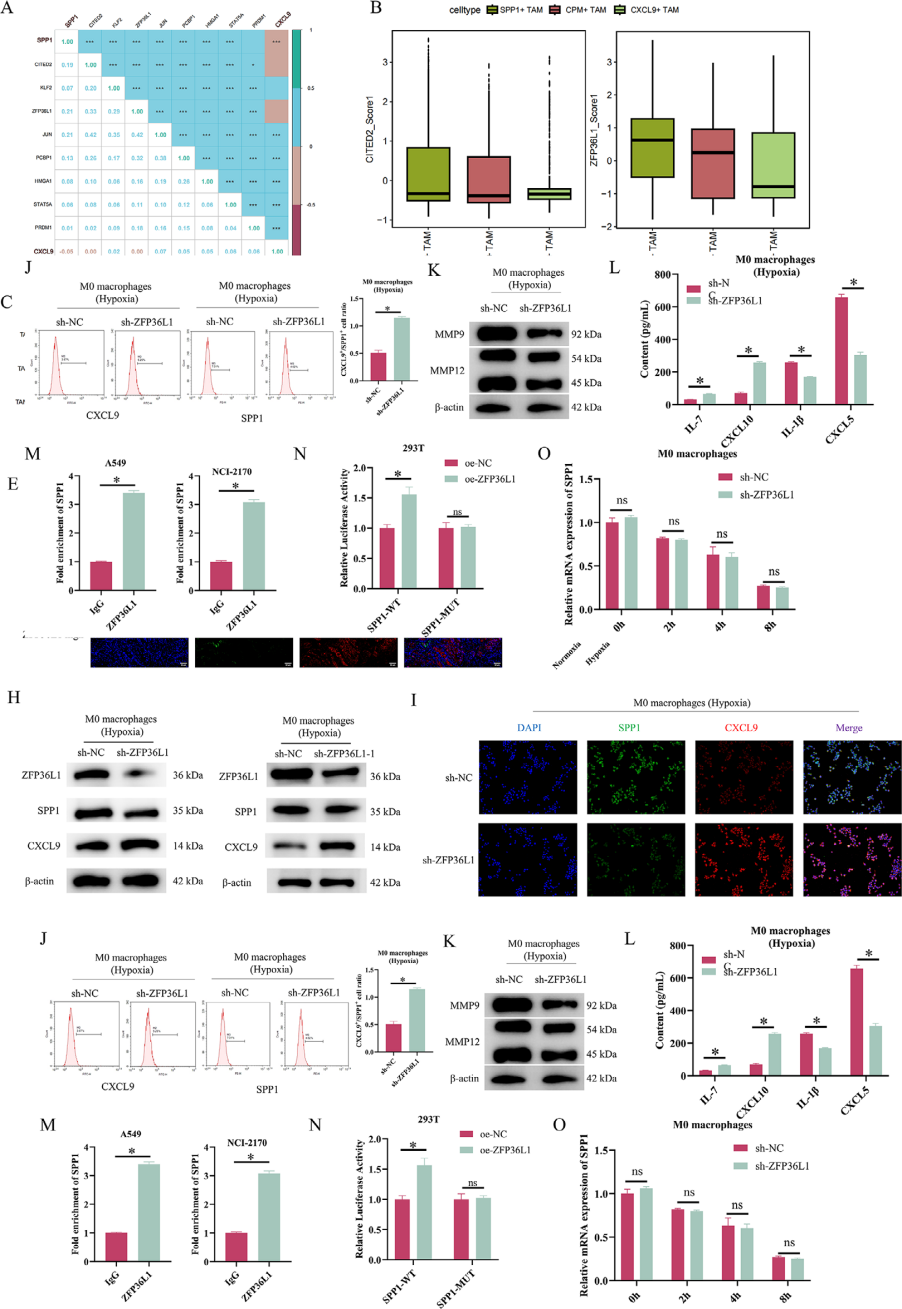
ZFP36L1 is a key gene regulating macrophage CXCL9:SPP1 ratio. (A) Correlation analysis between SPP1/CXCL9 and polarisation‐related transcription regulation factors. (B) Expression of CITED2 and ZFP36L1 in each tumour‐associated macrophage (TAM) subset. (C) Marker gene expression in each TAM subset. (D) Immunohistochemistry (IHC) analysis of ZFP36L1 and SPP1 expression. (E) Immunofluorescence analysis of ZFP36L1 and SPP1. (F) qRT‐PCR and (G) Western blotting (WB) of ZFP36L1 in M0 macrophages. ZFP36L1 knockdown M0 macrophages were established and cultured under hypoxia. (H) WB of ZFP36L1, SPP1 and CXCL9 protein expression. (I) Immunofluorescence staining assessing CXCL9 and SPP1 expression. (J) Flow cytometry of CXCL9^+^/SPP1^+^ ratio in macrophages. (K) WB of MMP9 and MMP12 protein expression. (L) ELISA of IL‐7, CXCL10, IL‐1β and CXCL5 levels. (M) ChIP assay validating ZFP36L1 binding to the SPP1 promoter. (N) Relative luciferase activity in ZFP36L1 overexpressing cells co‐transfected with SPP1 wild‐type (SPP1‐WT) or mutant (SPP1‐MUT) promoter constructs. (O) qRT‐PCR detection of SPP1 mRNA expression. ns, not significant; ^*^ *p* < .05.

To explore factors influencing the polarisation balance between SPP1^+^ and CXCL9^+^ TAMs, we queried the KnockTF 2.0 database for TFs of SPP1 or CXCL9. Using the ‘FindMarkers’ function, we identified marker genes for SPP1^+^ TAMs and CXCL9^+^ TAMs and intersected them with the TFs, yielding eight genes related to SPP1^+^ or CXCL9^+^ TAM polarisation. Correlation analysis confirmed a key overall linkage between the gene set and the SPP1/CXCL9 gene (Figure 3A). We selected the significantly differentially expressed CITED2 and ZFP36L1 for further analysis (Figure 3B). Gene expression patterns in the SPP1^+^, CXCL9^+^ and CPM^+^ TAM subsets are shown in Figure 3C. CPM^+^ TAMs expressed immune‐related genes (CPM, RARRES1, PLTP), SPP1^+^ TAMs upregulated genes associated with SPP1 induction (CITED2, ZFP36L1, MMP9 and MMP12), while CXCL9^+^ TAMs were characterised by chemokine genes such as CXCL10 and CCL5. ZFP36L1, known to suppress inflammatory mediators and exert regulatory effects under hypoxia,^18^, ^23^ showed substantial expression divergence between SPP1^+^ and CXCL9^+^ TAM subsets (Figure 3B). ZFP36L1 was thus selected for further investigation. To investigate the correlation between ZFP36L1 and CXCL9:SPP1 ratio and the OS and PFI of patients, we grouped the samples using the optimal cutoff and conducted survival curve analysis for OS and PFI. Figure S2A–D exhibits the Kaplan–Meier curves of OS and PFI for ZFP36L1 and CXCL9:SPP1 ratio. Patients with high expression of ZFP36L1 had a poorer prognosis, while those with a high CXCL9:SPP1 ratio had a better prognosis. In the GSE3141 dataset, we used the optimal cutoff for grouping and conducted survival curve analysis for OS. Figure S2E,F shows the OS curves of ZFP36L1 and CXCL9:SPP1 ratio, which are consistent with the results from TCGA database. Patients with high expression of ZFP36L1 had a poorer prognosis, while patients with high CXCL9:SPP1 ratio had a better prognosis. To explore the expression of ZFP36L1 and SPP1 in NSCLC tissues, we used IHC to divide the tissue samples into high and low expression groups of ZFP36L1. The IHC results also showed that compared to the ZFP36L1 low‐expression group, the expression level of SPP1 in the ZFP36L1 high‐expression group was increased (Figure 3D). IF staining experiments confirmed that in the ZFP36L1 high‐expression group, the co‐localisation signal of macrophage marker CD11b with ZFP36L1 significantly increased (Figure 3E).

M0 macrophages were cultured under normoxia or hypoxia. qRT‐PCR and WB showed that hypoxia upregulated ZFP36L1 in M0 macrophages (Figure 3F,G). We knocked down ZFP36L1 in THP‐1 cells and induced M0 macrophages, and further exposed them to a hypoxic environment. WB examined ZFP36L1, SPP1 and CXCL9 expression. ZFP36L1 knockdown downregulated SPP1 and upregulated CXCL9 (Figure 3H), which was validated by IF staining (Figure 3I). Flow cytometry showed that ZFP36L1 knockdown increased the CXCL9^+^/SPP1^+^ TAM ratio under hypoxia (Figure 3J). ZFP36L1 knockdown suppressed MMP9 and MMP12 protein expression (Figure 3K) and increased IL‐7 and CXCL10 levels while decreasing IL‐1β and CXCL5 levels (Figure 3L). We next investigated whether ZFP36L1 transcriptionally regulates SPP1. ChIP assays confirmed ZFP36L1 binding to the SPP1 promoter region (Figure 3M). Luciferase reporter assays showed that ZFP36L1 overexpression increased relative luciferase activity for the SPP1‐WT promoter but not the SPP1‐MUT promoter (Figure 3N). We also conducted an actinomycin D experiment to assess the post‐transcriptional stability of SPP1 mRNA. Silencing ZFP36L1 did not affect the stability of SPP1 mRNA in macrophages (Figure 3O). In conclusion, ZFP36L1 critically regulates the macrophage CXCL9:SPP1 ratio and promotes NSCLC progression under hypoxia by modulating SPP1 and CXCL9 expression.

### 3.4 ZFP36L1‐mediated regulation of macrophage CXCL9:SPP1 ratio influences NSCLC progression

**FIGURE 4:**
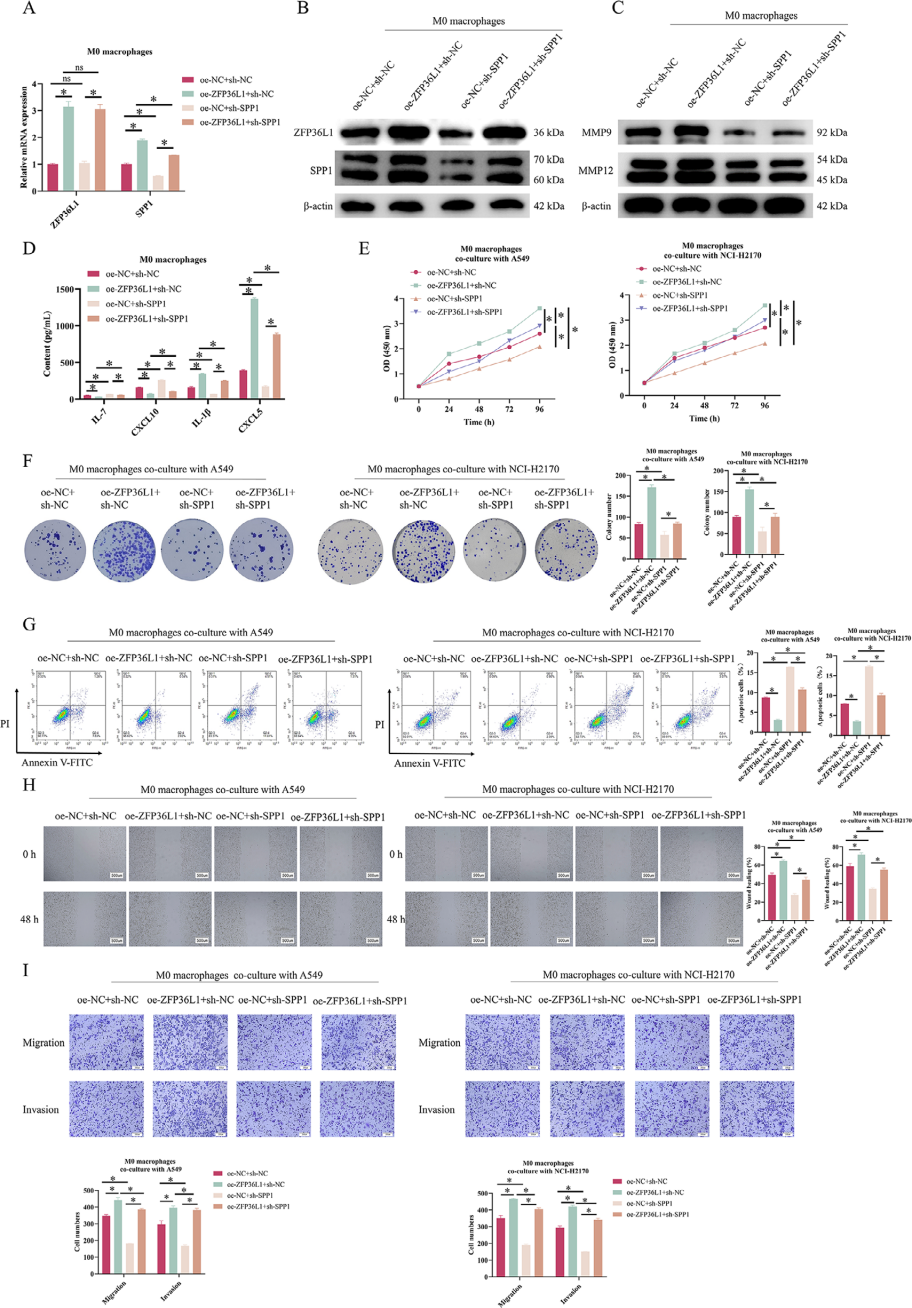
ZFP36L1 regulates macrophage CXCL9:SPP1 ratio to influence non‐small cell lung cancer (NSCLC) progression in the in vitro co‐culture system. M0 macrophages were divided into oe‐ZFP36L1 + sh‐NC, oe‐NC + sh‐SPP1, oe‐ZFP36L1 + sh‐SPP1 and oe‐NC + sh‐NC groups. (A) qRT‐PCR and (B) Western blotting (WB) of ZFP36L1 and SPP1 expression. M0 macrophages from each group were cultured under hypoxia. (C) WB of MMP9 and MMP12 protein expression. (D) ELISA of IL‐7, CXCL10, IL‐1β and CXCL5 levels. Hypoxic M0 macrophages from each group were co‐cultured separately with A549 or NCI‐H2170 cells. (E) CCK‐8 viability assay. (F) Colony formation assay for proliferation. (G) Apoptosis detection by flow cytometry. (H) Scratch migration assay. (I) Transwell migration and invasion assays. ns, not significant; ^*^ *p *< .05.

To probe into the impact of ZFP36L1 on macrophage CXCL9:SPP1 ratio and NSCLC progression, THP‐1 cells were divided into oe‐ZFP36L1 + sh‐NC, oe‐NC + sh‐SPP1, oe‐ZFP36L1 + sh‐SPP1 and oe‐NC + sh‐NC groups. qRT‐PCR and WB confirmed that ZFP36L1 overexpression significantly upregulated SPP1, which were partially counteracted by SPP1 knockdown (Figure 4A,B). Under hypoxia, the promotive effect of ZFP36L1 overexpression on MMP9 and MMP12 expression was evidently inhibited by SPP1 knockdown (Figure 4C). ELISA revealed that ZFP36L1 overexpression inhibited IL‐7 and CXCL10 secretion while promoting IL‐1β and CXCL5 levels; SPP1 knockdown produced opposite effects. ZFP36L1 overexpression could also reverse the effects of SPP1 knockdown on IL‐7, CXCL10, IL‐1β and CXCL5 (Figure 4D). Hypoxic M0 macrophages from the four groups were co‐cultured with A549 or NCI‐H2170 cells. In the co‐culture system, ZFP36L1 overexpression promoted tumour cell proliferation, migration and invasion, and inhibited apoptosis; SPP1 knockdown induced the opposite results. ZFP36L1 overexpression reversed the effects induced by SPP1 knockdown (Figure 4E–I).

Furthermore, we treated the M0 macrophages in the co‐culture system with SPP1 monoclonal antibody (HY‐P80771, MCE), and constructed the following groups: oe‐NC + IgG, oe‐ZFP36L1 + IgG, oe‐NC + SPP1a and oe‐ZFP36L1 + SPP1a. Simultaneously, hypoxia treatment was carried out. The treated M0 macrophages were then co‐cultured with A549 and NCI‐H2170 cells. ZFP36L1 overexpression enhanced tumour cells’ growth, migration and invasion abilities in the co‐culture system, while repressing cell apoptosis. However, the opposite results were observed after SPP1 monoclonal antibody treatment. These results indicate that the SPP1 monoclonal antibody reverses the tumour‐promoting effect caused by ZFP36L1 overexpression (Figure S3A–E).

**FIGURE 5:**
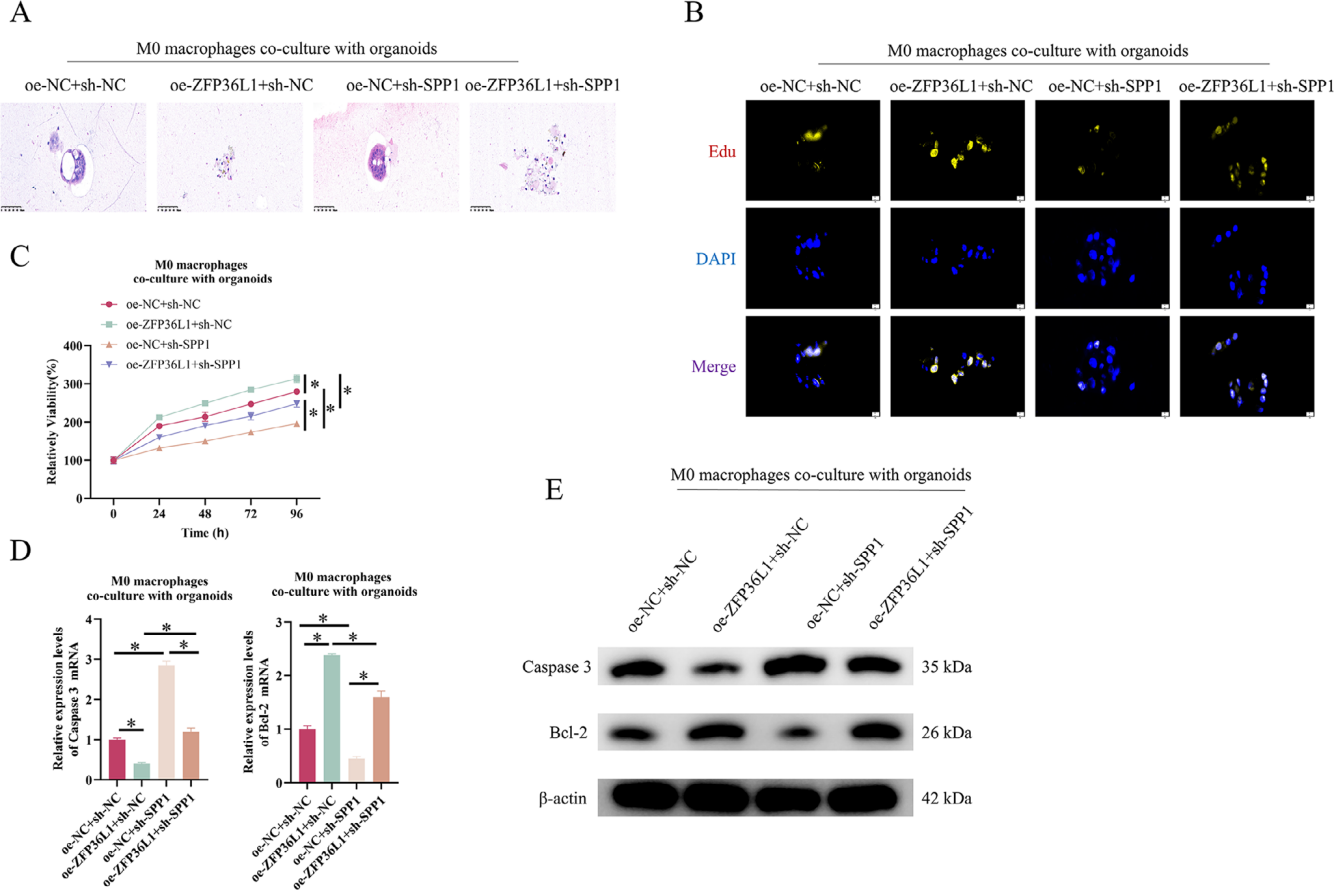
ZFP36L1 modulates macrophage CXCL9:SPP1 ratio to influence non‐small cell lung cancer (NSCLC) progression in a macrophage‐organoid co‐culture model. M0 macrophages were divided into oe‐ZFP36L1 + sh‐NC, oe‐NC + sh‐SPP1, oe‐ZFP36L1 + sh‐SPP1 and oe‐NC + sh‐NC groups. M0 macrophages were co‐cultured with patient‐derived organoids under hypoxia. (A) H&E staining showing pathological changes in organoids before and after treatment. (B) EdU staining assessing the proliferation of organoid cells. (C) CellTiter‐Glo assay for the viability of microtissues. (D) qRT‐PCR and (E) Western blotting (WB) of apoptotic Caspase 3 and Bcl‐2. ^*^ *p* < .05.

To validate the ZFP36L1‐SPP1 axis in NSCLC biologically, we co‐cultured the differentially treated macrophages with NSCLC patient‐derived organoids under hypoxic conditions. H&E staining revealed that ZFP36L1 overexpression induced extensive inflammatory infiltration, disrupted tissue architecture and cellular atrophy compared with the control group; SPP1 knockdown attenuated these pathological alterations. However, when ZFP36L1 was overexpressed in SPP1‐knockdown M0 macrophages, these pathological changes were reversed (Figure 5A). Moreover, EdU staining and CellTiter‐Glo assays demonstrated that SPP1 knockdown significantly suppressed the pro‑proliferative and viability‑enhancing effects induced by ZFP36L1 overexpression (Figure 5B,C). qRT‑PCR and WB analyses of apoptosis‑related proteins showed that ZFP36L1 overexpression upregulated the anti‑apoptotic Bcl‑2 and downregulated the pro‑apoptotic Caspase‑3; SPP1 knockdown resulted in the opposite outcome, which was reversed by overexpressing ZFP36L1 (Figure 5D,E). In conclusion, ZFP36L1 mediates macrophage CXCL9:SPP1 ratio by regulating SPP1 expression, thereby promoting NSCLC progression.

### 3.5 ZFP36L1‐driven SPP1^+^ TAM targets tumour cell surface CD44 to accelerate NSCLC progression

**FIGURE 6:**
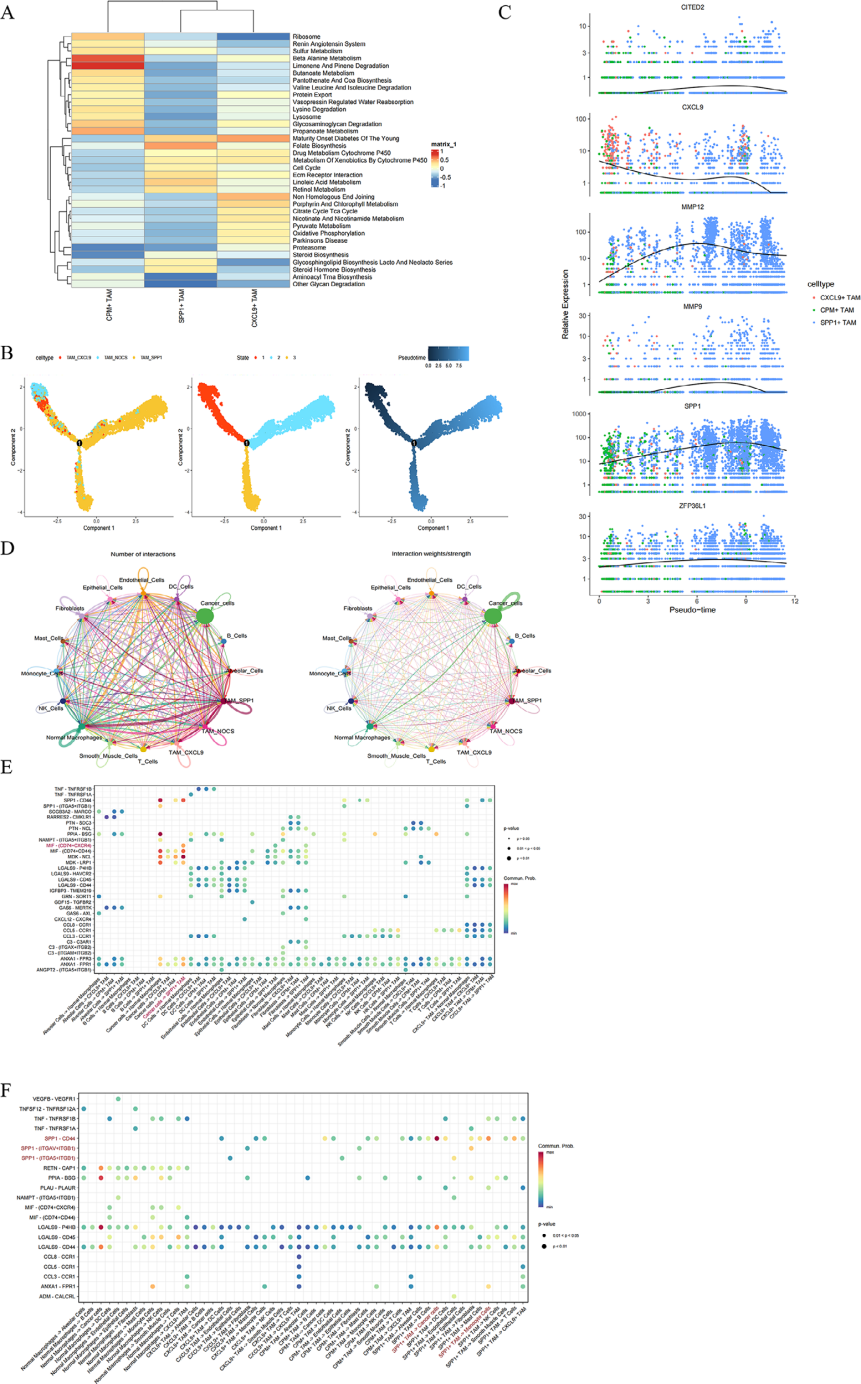
Impact of SPP1^+^ tumour‐associated macrophages (TAMs) on the non‐small cell lung cancer (NSCLC) microenvironment. (A) GSVA (KEGG) enrichment results of multiple TAM subsets. (B) Pseudotime analysis of TAM subsets. (C) Dynamic expression of ZFP36L1, CITED2, SPP1 and CXCL9 in TAM subsets during pseudotime. (D) Cell–cell communication of TAM subsets with other cellular tumour microenvironment (TME) components. Communication patterns of SPP1^+^ TAM as a signal sender (E) or a receiver (F) in a bubble plot.

GSVA (KEGG) pathway enrichment analysis revealed that SPP1^+^ TAMs significantly activated multiple pro‐tumourigenic pathways, notably the folate biosynthesis pathway associated with M2 polarisation and the sulphur metabolism pathway linked to anti‐inflammatory responses (Figure 6A). Pseudotime analysis indicated that under hypoxia, ZFP36L1 and CITED2 were upregulated in SPP1^+^ TAMs, promoting macrophage polarisation towards the SPP1^+^ phenotype (Figure 6B). SPP1 upregulation coincided with significant increases in MMP9 and MMP12, key effector molecules in NSCLC invasion and metastasis; CXCL9 expression showed a rise and fall trend in the pseudotime progression (Figure 6C). Expression patterns of genes collectively modulate NSCLC development. Differentiated cell–cell communication patterns between the three TAM subsets (CPM^+^, SPP1^+^ and CXCL9^+^) and 12 other cell types were revealed by CellChat (Figure 6D). SPP1^+^ TAM, as a signal receiver, engaged in cell–cell communication through the MIF‐(CD74 + CXCR4) ligand–receptor pair (Figure 6E); as a signal sender, it primarily interacted with various integrin receptors, including CD44, ITGAV + ITGB1 and ITGA5 + ITGB1 (Figure 6F).

**FIGURE 7:**
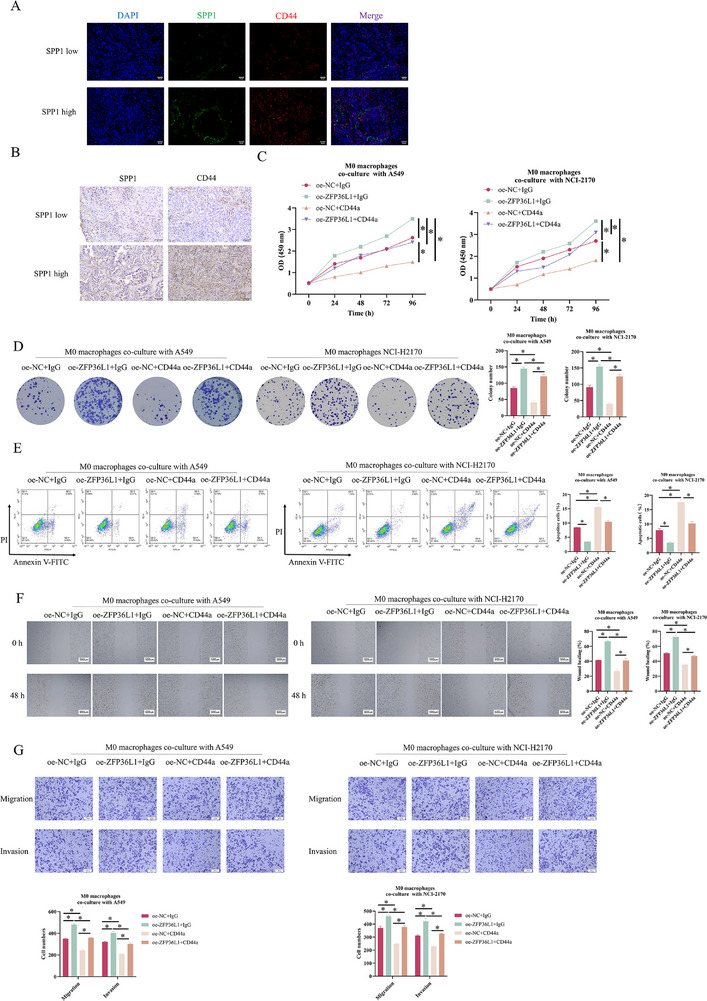
ZFP36L1‐driven SPP1^+^ tumour‐associated macrophage (TAM) accelerates non‐small cell lung cancer (NSCLC) progression by binding to CD44 on tumour cells. (A) Immunohistochemistry (IHC) analysis of SPP1 and CD44 expression. (B) Immunofluorescence analysis of SPP1 and CD44. CD44 monoclonal antibody was introduced into a co‐culture system of M0 macrophages with A549 or NCI‐H2170 cells under hypoxic conditions. Experimental groups included: oe‐NC + IgG, oe‐ZFP36L1 + IgG, oe‐NC + CD44a and oe‐ZFP36L1 + CD44a. (C) CCK‐8 viability assay. (D) Colony formation assay for proliferation. (E) Flow cytometry detecting apoptosis. (F) Scratch migration assay. (G) Transwell migration and invasion assays. ^*^ *p* < .05.

To investigate SPP1 and CD44 expression in NSCLC tissues, we sub‐grouped the tissue samples. IF staining confirmed that in the SPP1 high‐expression group, the levels of SPP1 and CD44 significantly increased (Figure 7A). The IHC demonstrated that compared to the SPP1 low‐expression group, CD44 was higher in the SPP1 high‐expression group (Figure 7B). Given that SPP1^+^ TAM can secrete SPP1 that binds to CD44, a well‐characterised surface marker on tumour cells,^24^, ^25^ we hypothesised that SPP1^+^ TAM promotes NSCLC through CD44. Using a CD44 monoclonal antibody (HY‐P80062, MCE) in the co‐culture system of M0 macrophage and A549 or NCI‐H2170 cells under hypoxia, we evaluated the malignancy of tumour cells in the following groups: oe‐NC + IgG, oe‐ZFP36L1 + IgG, oe‐NC + CD44a and oe‐ZFP36L1 + CD44a, through CCK‐8, colony formation, Transwell and scratch assays and flow cytometry. Compared to the control, anti‐CD44 treatment alone significantly attenuated ZFP36L1 overexpression‐induced enhancement of proliferation, migration, invasion and anti‐apoptosis (Figure 7C–G). In conclusion, ZFP36L1 accelerates NSCLC progression by promoting the SPP1^+^ phenotype and increasing SPP1 to bind to CD44 on tumour cells.

### 3.6 In vivo, ZFP36L1 knockout in macrophages inhibits NSCLC progression by balancing CXCL9:SPP1 ratio

**FIGURE 8:**
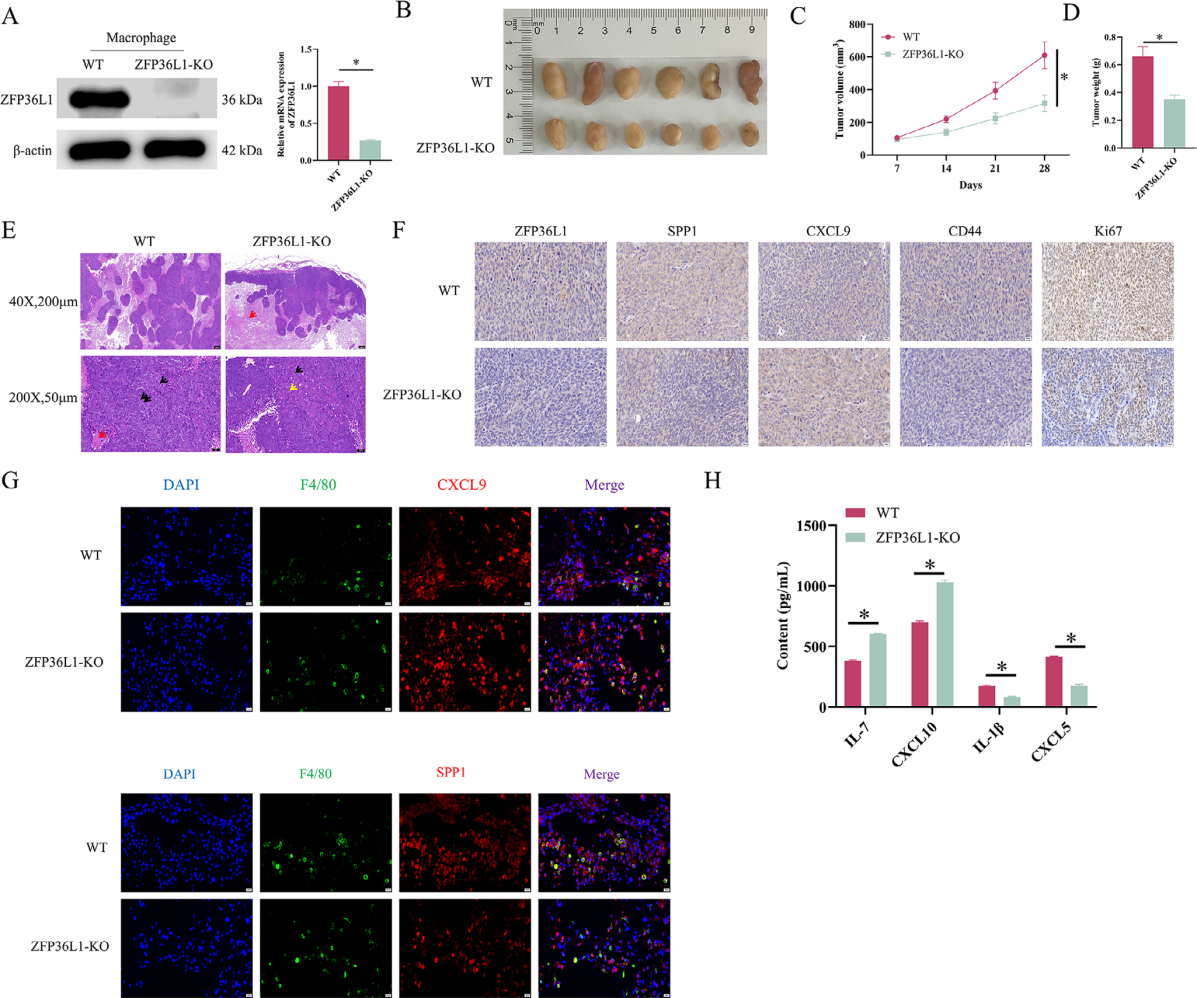
ZFP36L1 knockout in macrophage inhibits non‐small cell lung cancer (NSCLC) progression by balancing CXCL9:SPP1 ratio in vivo. Mouse models with conditional ZFP36L1 knockout in macrophages were established. (A) qRT‐PCR and Western blotting (WB) analyses of ZFP36L1 expression. (B) Representative images of mouse tumours. (C) Tumour volume. (D) Tumour weight. (E) Histopathological test of tumour tissues by H&E staining. (F) Immunohistochemistry (IHC) analysis of ZFP36L1, SPP1, CXCL9, CD44 and Ki67 expression. (G) Immunofluorescence co‐localisation of F4/80‐positive cells with CXCL9 or SPP1. (H) ELISA quantification of IL‐7, CXCL10, IL‐1β and CXCL5 levels. ^*^ *p* < .05.

In vitro, ZFP36L1 promotes NSCLC progression by modulating macrophage CXCL9:SPP1 ratio. To test this in vivo, we generated a mouse model with conditional knockout of ZFP36L1 in macrophages. Tumour growth was monitored and macrophages were isolated from mouse tissues. qRT‐PCR and WB confirmed the absence of ZFP36L1 expression in the ZFP36L1‐KO group (Figure 8A). ZFP36L1 knockout repressed tumour volume and weight, compared to the WT (Figure 8B–D). H&E staining revealed distinct pathological changes in mouse tumour tissues. WT tissues showed solid sheets of highly differentiated, spindle‐ or oval‐shaped tumour cells, with evident localised nuclear division (black arrows) and large, well‐demarcated necrotic areas containing abundant proteinaceous fibrin from necrotic cells (red arrow). ZFP36L1‐KO tissues showed widespread necrosis with clear boundaries and abundant proteinaceous fibrin (red arrow); the remaining tumour parenchyma was peripherally concentrated, with unclear, occasional mitotic figures (black arrows) and mild degenerative necrosis (yellow arrow); the stroma showed no identifiable fibroblastic septa (Figure 8E). IHC analysis showed that ZFP36L1 knockout significantly downregulated SPP1, CD44 and Ki67 protein expression and upregulated CXCL9 (Figure 8F). IF confirmed increased co‐localisation of the macrophage marker F4/80 with CXCL9 and decreased co‐localisation with SPP1 in the ZFP36L1‐KO group (Figure 8G). ELISA indicated that ZFP36L1 deficiency significantly increased IL‐7 and CXCL10 levels and decreased IL‐1β and CXCL5 in the TME (Figure 8H). In conclusion, ZFP36L1 knockout in macrophages suppresses NSCLC progression in vivo by modulating CXCL9:SPP1 ratio balance.

## 4 DISCUSSION

NSCLC is a malignancy with high incidence and mortality, whose significant tumour heterogeneity frequently results in suboptimal treatment outcomes.^26^ Exploring novel molecular biomarkers is therefore of great importance. In recent years, scRNA‐seq analysis has attracted attention in various malignant tumours, such as pancreatic ductal adenocarcinoma,^27^ nasopharyngeal carcinoma^28^ and NSCLC,^21^ providing new insights into how immune cells within the TME influence malignant progression. The scRNA‐seq was used to explore the function of the macrophage CXCL9:SPP1 ratio in NSCLC. Building on this, we combined cell and animal experiments to demonstrate that ZFP36L1 promotes the secretion of SPP1 by increasing the proportion of SPP1^+^ TAMs, which in turn binds to CD44 on tumour cells, ultimately driving malignant progression. These findings define ZFP36L1's role in NSCLC and establish it as a therapeutic target, offering a theoretical basis for macrophage polarisation‐based intervention strategies.

The functional heterogeneity of the TME is determined by its TAM polarisation. Pro‐inflammatory M1 TAMs exert anti‐tumour effects by releasing pro‐inflammatory factors and reactive oxygen species. Anti‐inflammatory M2 TAMs, on the other hand, drive tumour progression by releasing growth factors and immunosuppressive cytokines.^29^ A distinct SPP1‐high TAM subset (SPP1^+^ TAMs) tightly linked to tumour necrosis was identified.^30^ SPP1^+^ TAMs are considered to be pro‐tumourigenic in various cancers, such as colorectal cancer,^30^ HCC^22^ and NSCLC, and are linked to poor prognosis. Recent scRNA‐seq analyses have revealed a mutual exclusivity in CXCL9 and SPP1 expression in macrophages, with their ratio determining whether macrophages exhibit pro‐ or anti‐tumour phenotypes.^15^ Gu et al.^31^ identified the CXCL9:SPP1 ratio as a prognostic predictor for HCC. Consistently, our scRNA‐seq analysis found that SPP1^+^ TAMs constitute the largest proportion among macrophage subpopulations and are associated with NSCLC progression. This suggests that modulating the CXCL9:SPP1 ratio in TAMs could represent a novel therapeutic direction, although the underlying mechanisms require further exploration.

Hypoxia promotes the generation of SPP1^+^ TAMs to promote cancer malignant progression.^32^ Hypoxia is a key factor in the TME that drives malignant progression by influencing metabolic reprogramming, angiogenesis and tumour immunity.^33^, ^34^ In NSCLC, hypoxia can significantly promote tumour cell migration and invasion.^35^ Additionally, hypoxia participates in tumourigenesis and development by regulating the expression of specific CXC chemokines in the TME.^36^ For instance, HIF‐1α transcriptionally suppresses chemokines (CXCL9, CXCL10 and CXCL11), and reduces CD8^+^ T‐cell infiltration in the TME of colorectal cancer, thereby promoting tumour immune evasion and malignant progression.^37^ Similarly, in our study, hypoxia downregulated CXCL9 and CXCL10 in macrophages while upregulating CXCL5 and SPP1, leading to a significant decrease in the CXCL9:SPP1 ratio in macrophages. Co‐culture of hypoxic macrophages with NSCLC cells promoted malignant cancer phenotypes. This indicates that hypoxia drives a decrease in the macrophage CXCL9:SPP1 ratio, inducing NSCLC progression. Therefore, identifying key factors in the TME that drive macrophage polarisation towards the SPP1^+^ phenotype is crucial.

We identified ZFP36L1 as a key target gene influencing CXCL9:SPP1 ratio through scRNA‐seq. ZFP36L1 downregulates inflammatory factors and exerts regulatory functions in hypoxic environments.^18^, ^23^ Notably, ZFP36L1 overexpression is documented in gastric cancer tissues and closely correlates with worse prognosis, suggesting its oncogenic role.^16^ However, the specific impact of ZFP36L1 on NSCLC progression under hypoxia had not been clearly established until our research. By co‐culturing hypoxic macrophages with A549 or NCI‐H2170 cells, we demonstrated that ZFP36L1 overexpression promoted tumour cell growth, migration and invasion, and inhibited apoptosis, confirming its tumour‐promoting effects. Two studies on lung cancer have reported the tumour suppressor role of ZFP36L1. The first study found that ZFP36L1 inhibited LUAD cells’ proliferation, survival and cycle progression.^38^ The second study showed that the R9‐ZFP36L1 fusion protein could significantly inhibit tumour growth in nude mice and lower the level of various angiogenic and inflammatory cytokines.^39^ The results of this study are contrary to previous studies. We hypothesise the reasons as follows. Previous studies were mostly conducted under normoxia conditions, while this study simulated the hypoxic characteristics of the TME (1% O_2_). The hypoxic environment may change the metabolic state and gene expression profile of macrophages, reshape the target recognition ability of ZFP36L1, and lead to the promotion of the malignant progression of tumour cells. Cell–cell communication analysis revealed the binding of SPP1 and CD44 on the tumour cell surface when SPP1^+^ TAMs act as the sender. CD44 is a non‐kinase cell surface transmembrane glycoprotein, whose upregulation can promote malignant tumour progression.^25^ In the co‐cultivation system of macrophages with NSCLC cells under hypoxia, we revealed that adding the CD44 monoclonal antibody inhibited the malignant phenotype of the tumour cells. Notably, knocking down ZFP36L1 reversed these effects. In conclusion, ZFP36L1 promotes macrophage polarisation towards the SPP1^+^ phenotype, accelerating NSCLC progression by binding to CD44 on tumour cells. This potentially offers a new research direction for overcoming the immunosuppressive TME in NSCLC. The preliminary ChIP and dual luciferase experiments in this study are not sufficient to distinguish direct transcriptional activation from indirect regulatory mechanisms, and knocking down ZFP36L1 does not affect the stability of SPP1 mRNA in macrophages. Based on these findings, we propose two possible regulatory hypotheses: (1) ZFP36L1 may degrade the mRNA of a repressive transcription factor, thereby relieving its inhibitory effect on the SPP1 promoter and indirectly upregulating SPP1 expression; (2) ZFP36L1 may interact with non‐coding RNAs (such as lncRNAs) or transcription machinery components and be recruited to the SPP1 promoter region to participate in regulation. However, the specific molecular mechanism between ZFP36L1 and SPP1 still needs to be further elucidated. In subsequent studies, we plan to use RNA immunoprecipitation sequencing technology to identify the genome‐wide RNA binding targets of ZFP36L1 in hypoxic macrophages, and use CRISPR‐Cas9 technology to edit the potential ZFP36L1 binding motifs in the SPP1 promoter region for function verification.

In summary, ZFP36L1 is a key regulator of macrophage CXCL9:SPP1 ratio in NSCLC progression. Through cellular experiments and animal model validation, we confirmed that hypoxia‐induced upregulation of ZFP36L1 promotes NSCLC progression by decreasing the CXCL9:SPP1 ratio in macrophages (Graphical Abstract). However, certain limitations should be acknowledged. First, bioinformatic analysis had a limited sample size, with scRNA‐seq data derived from only four control samples and 26 NSCLC samples. Additionally, the function of the SPP1–CD44 interaction in vivo has not been validated. Given the core innovation points of the current research, we did not conduct a systematic and comprehensive verification of the upstream hypoxia regulatory mechanism of ZFP36L1 and the various downstream signaling pathways of SPP1–CD44. Sample size should be expanded in future research to validate the SPP1–CD44 axis in NSCLC progression in vivo. We plan to systematically identify the upstream hypoxia mechanism of ZFP36L1, the specific downstream pathways activated by SPP1–CD44, and their contribution in NSCLC through phosphorylation protein chips, RNA‐seq and functional rescue experiments.

## AUTHOR CONTRIBUTIONS

*Conceptualisation, methodology, investigation, data curation, visualisation and writing—original draft*: Lijie Wang. *Software, formal analysis, validation and data curation*: Biao Chen. *Investigation, resources, data curation and supervision*: Jinxian He. *Investigation, methodology, experiments and data acquisition*: Chengbin Lin. *Validation, formal analysis and writing—review and editing*: Weiyu Shen. *Resources, funding acquisition, project administration and writing—review and editing*: Wang Lv. *Conceptualisation, resources, supervision, funding acquisition and writing—review and editing*: Luming Wang. *Conceptualisation, methodology, resources, supervision, funding acquisition, project leadership and writing—review and editing*: Jian Hu.

## CONFLICTS OF INTEREST STATEMENT

The authors declare they have no conflicts of interest.

## ETHICS STATEMENT

This study was approved by the Ethics Committee of Ningbo Medical Center Lihuili Hospital(approval number KY2025PJ501). The animal study protocol was approved by the Ethics Committee of Zhejiang Luoxi Medical Technology Co., Ltd., Hangzhou, China (approval number LX4825021301).

## CONSENT TO PARTICIPATE

Informed consent was obtained from all participants.

## Supporting information



Supporting information

Supporting information

Supporting information

Supporting information

Supporting information

Supporting information

## Data Availability

The data supporting the current study are available from the corresponding author upon reasonable request.
