# Supplementary figures and images for: ZFP36L1 promotes non‐small cell lung cancer progression under hypoxia by modulating CXCL9:SPP1 polarity: A single‐cell transcriptomic study
Source: Clin Transl Med. 2026 Mar 29;16(4):e70642. doi: 10.1002/ctm2.70642 (PMC13140207; doi:10.1002/ctm2.70642)

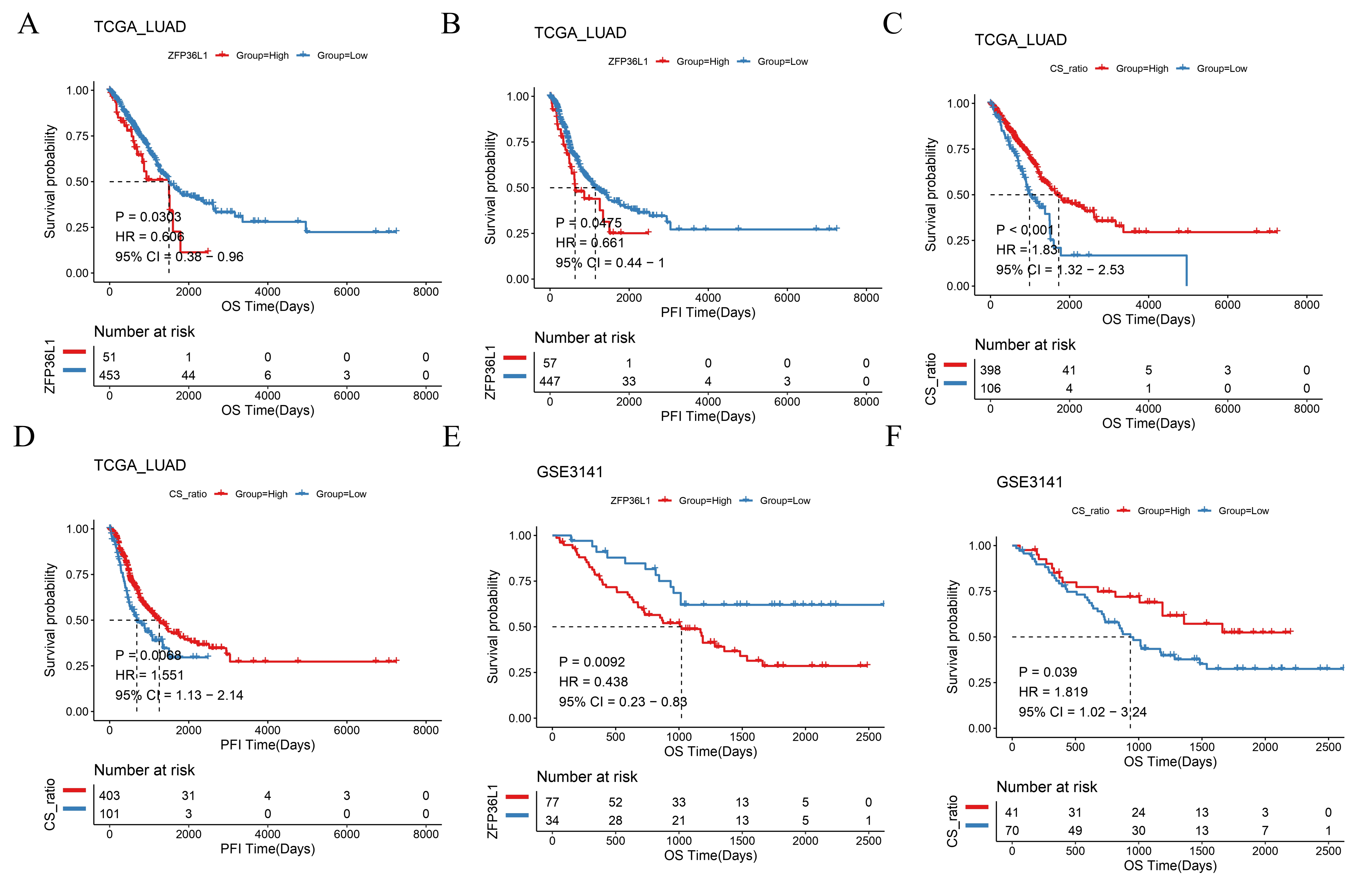

Supplement: Supplementary file 2 — Supporting information [file CTM2-16-e70642-s005.TIF]

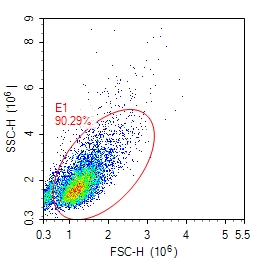

Supplement: Supplementary file 5 — Supporting information [file CTM2-16-e70642-s006.zip › Supplementary materials/Figure 2D-CXCL9-KB/1.jpg]

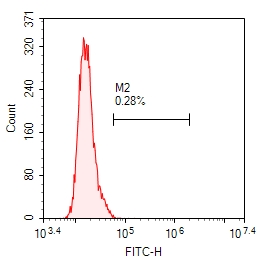

Supplement: Supplementary file 5 — Supporting information [file CTM2-16-e70642-s006.zip › Supplementary materials/Figure 2D-CXCL9-KB/2.jpg]

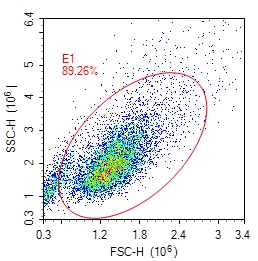

Supplement: Supplementary file 5 — Supporting information [file CTM2-16-e70642-s006.zip › Supplementary materials/Figure 2D-SPP1-KB/1.jpg]

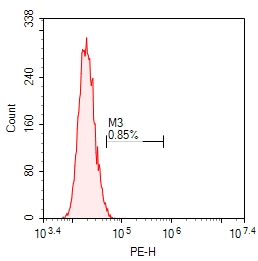

Supplement: Supplementary file 5 — Supporting information [file CTM2-16-e70642-s006.zip › Supplementary materials/Figure 2D-SPP1-KB/2.jpg]

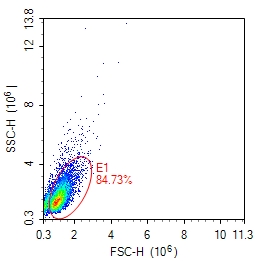

Supplement: Supplementary file 5 — Supporting information [file CTM2-16-e70642-s006.zip › Supplementary materials/Figure 3J-CXCL9-KB/1.jpg]

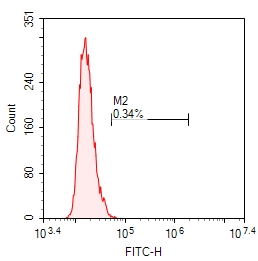

Supplement: Supplementary file 5 — Supporting information [file CTM2-16-e70642-s006.zip › Supplementary materials/Figure 3J-CXCL9-KB/2.jpg]

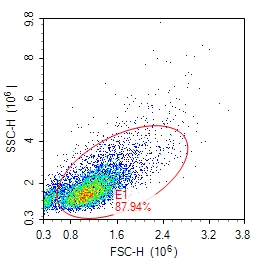

Supplement: Supplementary file 5 — Supporting information [file CTM2-16-e70642-s006.zip › Supplementary materials/Figure 3J-SPP1-KB/1.jpg]

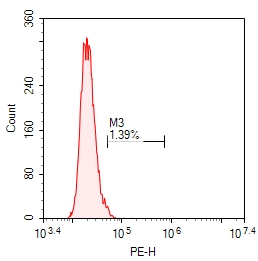

Supplement: Supplementary file 5 — Supporting information [file CTM2-16-e70642-s006.zip › Supplementary materials/Figure 3J-SPP1-KB/2.jpg]
