# Supplementary material for: ZFP36L1 promotes non‐small cell lung cancer progression under hypoxia by modulating CXCL9:SPP1 polarity: A single‐cell transcriptomic study
Source: Clin Transl Med. 2026 Mar 29;16(4):e70642. doi: 10.1002/ctm2.70642 (PMC13140207; doi:10.1002/ctm2.70642)
Supplement: Supplementary file 4 — Supporting information [file CTM2-16-e70642-s001.docx]

Manuscript Title: ZFP36L1 Promotes Non-Small Cell Lung Cancer Progression Under Hypoxia by Modulating CXCL9:SPP1 Polarity: A Single-Cell Transcriptomic Study

Lijie Wang (a, b), Biao Chen (b), Jinxian He (b), Chengbin Lin (b), Weiyu Shen (b), Wang Lv (a), Luming Wang (a), Jian Hu (a)

a Department of Thoracic Surgery, The First Affiliated Hospital, Zhejiang University School of Medicine, Hangzhou, Zhejiang, China.

b Department of Thoracic Surgery, The Affiliated Lihuili Hospital of Ningbo University, Ningbo, Zhejiang, China.

Co-corresponding author: Luming Wang (a), E-mail: 1507144@zju.edu.cn.

Corresponding author: Jian Hu, Department of Thoracic Surgery, The First Affiliated Hospital, Zhejiang University School of Medicine, Hangzhou, Zhejiang, China.

Adress: No. 79, Qingchun Road, Shangcheng District, Hangzhou, Zhejiang, 310003, China. E-mail: dr_hujian@zju.edu.cn.

**Supplementary Fig. 1 Cell clustering and annotation of NSCLC cells based on scRNA-seq.**

**A:** UMI counts (nCount), number of detected genes (nFeature), and mitochondrial gene percentage before quality control. **B:** Point density plots of mitochondrial gene percentage versus nCount (left), nFeature versus nCount (right) before quality control. **C:** nCount, nFeature, and mitochondrial gene percentage after filtering. **D:** Point density plots of mitochondrial gene percentage versus nCount (left), nFeature versus nCount (right) after quality control. **E:** Variation in highly variable gene expression. **F:** Principal component analysis results. **G:** Principal component analysis of macrophages. **H:** UMAP visualization of macrophage subpopulations. **I:** Annotation of macrophage subpopulations.

**Supplementary Fig. 2**

1. **F:** Comparison of the correlation between the ZFP36L1 and CXCL9:SPP1 ratio with the OS and PFI of the patients.

**Supplementary Fig. 3 ZFP36L1 regulates macrophage CXCL9:SPP1 ratio to influence NSCLC progression in the *in vitro* co-culture system.**

M0 macrophages were divided into oe-NC+IgG, oe-ZFP36L1+IgG, oe-NC+SPP1a, and oe-ZFP36L1+SPP1a groups. **A:** CCK-8 viability assay. **B:** Colony formation for proliferation. **C:** Apoptosis detection by flow cytometry. **D:** Scratch migration assay. **E:** Transwell migration and invasion assays. ns, not significant; **p* < 0.05.
