# Supplementary material for: ZFP36L1 promotes non‐small cell lung cancer progression under hypoxia by modulating CXCL9:SPP1 polarity: A single‐cell transcriptomic study
Source: Clin Transl Med. 2026 Mar 29;16(4):e70642. doi: 10.1002/ctm2.70642 (PMC13140207; doi:10.1002/ctm2.70642)
Supplement: Supplementary file 6 — Supporting information [file CTM2-16-e70642-s002.pdf]

iThenticate®

Manuscript-----.docx
Feb 14, 2026
9291 words / 54197 characters

Manuscript-----

Sources Overview

14%
OVERALL SIMILARITY

#	Source	Type	Similarity
1	www.frontiersin.org	INTERNET	2%
2	www.mdpi.com	INTERNET	<1%
3	www.ncbi.nlm.nih.gov	INTERNET	<1%
4	link.springer.com	INTERNET	<1%
5	www.dovepress.com	INTERNET	<1%
6	Jiaqi Li, Chaozhe Ma, Pei Cao, Weihua Guo et al. "A CD147-targeted small-molecule inhibitor potentiates gemcitabine efficacy by trigg...	CROSSREF	<1%
7	rupress.org	INTERNET	<1%
8	assets-eu.researchsquare.com	INTERNET	<1%
9	molmed.biomedcentral.com	INTERNET	<1%
10	www.fedoa.unina.it	INTERNET	<1%
11	Yiding Liu, Bing Yan, Hui Dong, Yi Li, Yuying Zhang, Yi Wang, Ni Kou, Lu Gao. "P4HA1 facilitates SPP1+ tumor-associated macrophage...	CROSSREF	<1%
12	www.coursehero.com	INTERNET	<1%
13	Kai Chen, Qi Wang, Mingzhe Li, Huahu Guo, Weikang Liu, Feng Wang, Xiaodong Tian, Yinmo Yang. "Single-cell RNA-seq reveals dynami...	CROSSREF	<1%
14	pmc.ncbi.nlm.nih.gov	INTERNET	<1%
15	topsecretapiaccess.dovepress.com	INTERNET	<1%
16	Tianfei Zhong, Ying Li, Meng Jin, Jingqun Liu, Zhenyu Wu, Feiye Zhu, Lisha Zhao, Yongsheng Fan, Li Xu, Jinjun Ji. "Downregulation of 4...	CROSSREF	<1%

17 Yongzhong Wu. "The zinc finger repressor, ZBP-89, recruits histone deacetylase 1 to repress vimentin gene expression", Genes to Cell... <1%
CROSSREF

18 jeccr.biomedcentral.com <1%
INTERNET

19 www.aging-us.com <1%
INTERNET

20 Chengquan Shen, Jing Liu, Ding Hu, Changxue Liu, Fei Xie, Yonghua Wang. "Tumor-intrinsic ENO1 inhibition promotes antitumor immu... <1%
CROSSREF

21 files01.core.ac.uk <1%
INTERNET

22 genomebiology.biomedcentral.com <1%
INTERNET

23 public-pages-files-2025.frontiersin.org <1%
INTERNET

24 Jiayun Chen, Wenmin Lu, Yanni Lou, Jing Liu et al. "Integrating single cell- and spatial- resolved transcriptomics unravels the inter-tum... <1%
CROSSREF

25 O. G. Avwioro, C. P. Aloamaka, O. B. Olabampe, T. Oduola. " Collagen and muscle stain obtained from ", Scandinavian Journal of Clinic... <1%
CROSSREF

26 bmcmedicine.biomedcentral.com <1%
INTERNET

27 cancerci.biomedcentral.com <1%
INTERNET

28 worldwidescience.org <1%
INTERNET

29 www.nature.com <1%
INTERNET

Preprint source
30 www.researchsquare.com <1%
INTERNET

31 Chenggen Gao, Jintao Wu, Fangyan Zhong, Xianxin Yang, Hanwen Liu, Junming Lai, Jing Cai, Weimin Mao, Huijuan Xu. "Integrative an... <1%
CROSSREF

32 Masha Huang, Jingyi Sun, Qingqing Jiang, Xin Zhao, Hanxue Huang, Mengrong Lei, Shilong Jiang, Fuqiang Yuan, Zhaoqian Liu. "CircKI... <1%
CROSSREF

33 www.1stoncology.com <1%
INTERNET

34 www.research-collection.ethz.ch <1%
INTERNET

35 Lkhagva-Ochir Tovuu, Satoru Imura, Tohru Utsunomiya, Yuji Morine et al. "Role of CD44 expression in non-tumor tissue on intrahepatic... <1%
CROSSREF

36 Yong Xi, Zhijie Zhao, Yundong Zhou, Chengliang Yin et al. "Macrophage efferocytosis mediated by the TP63-RAC2 pathway promotes i... <1%
CROSSREF

37 Zhichao Li, Yuanjie Zhong, Dan Ye, Jincheng Yang, Linbao Chen. "Revealing NAPSA's role in ccRCC: Insights from single-cell RNA sequ... <1%
CROSSREF

38 jitc.bmj.com <1%
INTERNET

39 J. M. Oldhoff. "Atopy patch test in patients with atopic eczema/dermatitis syndrome: comparison of petrolatum and aqueous solution... <1%
CROSSREF

40 Qi Liu, Liusheng Wu, Meiling Lu, Hao Jia, Xiaoqiang Li. "Identification of the Immune Microenvironment, Mutation Burden, Immunother... <1%
CROSSREF

41 biologydirect.biomedcentral.com <1%
INTERNET

42 stemcellres.biomedcentral.com <1%
INTERNET

43 translational-medicine.biomedcentral.com <1%
INTERNET

44 www.degruyter.com <1%
INTERNET

45 www.hindawi.com <1%
INTERNET

46 "The Fine Blueprint of Integrating Immunotherapy and Radiotherapy", Springer Science and Business Media LLC, 2025 <1%
CROSSREF

47 Chundong Liu, Zeguang Chen. "ZC3H13 knockdown enhances the inhibitory effect of sevoflurane on gastric cancer cell malignancy b... <1%
CROSSREF

48 Dong Chang, Daolong Li, Yuxi Sun, Jiekang Shi, Shengping Zhang, Chuangui Wang. "Targeting Lactylation for Cancer: Mechanisms, Eff... <1%
CROSSREF

49 Fanshu Li, Dafeng Xu, Zhen Tang, Yangfeng Lai, Qiumeng Liu, Huifang Liang, Hanhua Dong, Jia Song. "SPP1+ Macrophages and the O... <1%
CROSSREF

50 Guangxian Mao, Jixian Liu. "CALML3-AS1 enhances malignancies and stemness of small cell lung cancer cells through interacting wi... <1%
CROSSREF

51 Hao Xie, Zhaoying Wu, Zhenhan Li, Yong Huang, Junwei Zou, Hailang Zhou. "Significance of ZEB2 in the immune microenvironment of... <1%
CROSSREF

52 Juanjuan Wang, Yi Shi, Yunhuan Gao, Ningning Zhu, Yuqing Liu, Yuan Zhang, Chen Xu, Rongcun Yang. "Targeting macrophage-derived ... <1%
CROSSREF

53 Kaiyan Qiu, Zhixiong Zheng, Yingfu Huang. "Long intergenic noncoding RNA 00844 promotes apoptosis and represses proliferation of... <1%
CROSSREF

54 Kondo, Misaki. "HER2-Targeted Theranostic Radioimmunoconjugates for SPECT Imaging and α-Particle Radioimmunotherapy of HER... <1%
PUBLICATION

55 Lei Xu, Haoyong Kuang, Haodong Peng, Sen Wu, Yu Bai, Xiangbo Jia, Wenjian Yao. "A LINC00472-encoded polypeptide impedes migra... <1%
CROSSREF

56 Miho Akimoto, Takao Susa, Noriyuki Okudaira, Nobuko Koshikawa et al. "Hypoxia induces downregulation of the tumor-suppressive s... <1%
CROSSREF

57 Qiuju Miao, Nan Jiang, Qinmeng Yang, Ismail Mohamed Hussein, Zhen Luo, Lei Wang, Shenyu Yang. "Multi-stage controllable degrada... <1%
CROSSREF

58 Ren, Jianlan. "Computational Methods for Single-Cell and Multi-Omic Data Integration and Regulatory Network Inference.", New Jerse... <1%
PUBLICATION

59 Ye Wu, Ling Zhang, Wenhui Li, Dong Yan, Jingjing Yue, Zhusheng Liu. "Hsa_circ_0071271 affected the progression of non-small cell lu... <1%
CROSSREF

60 Yifan Xu, Chonghui Zhang, Jinpeng Wu, Mingyang Qiu, Meng Zhu, Chao Wang, Yugong Feng. "Integrating single-cell RNA sequencing a... <1%
CROSSREF

61 Zehua He, Qingfeng Chen, Wanrong He, Junyue Cao, Shunhan Yao, Qingqiang Huang, Yu Zheng. "Hepatocellular carcinoma subtypes ... <1%
CROSSREF

62 Zheng Liu, Mengmeng Ji, Fangtong Jin, Feng Jiang, Xiaoping Liu. "Expression and clinical significance of miR-17-5p in tumor tissues ... <1%
CROSSREF

63 journals.lww.com
INTERNET <1%

64 www.ijbs.com
INTERNET <1%

65 Parteek Prasher, Mousmee Sharma, Indu Pal Kaur, Ronan MacLoughlin, Raimar Loebenberg, Neal M. Davies, Kamal Dua. "Nanotechno...
PUBLICATION <1%

66 Topi A. Tervonen. "Overexpression of a truncated TrkB isoform increases the proliferation of neural progenitors", European Journal of ...
CROSSREF <1%

67 Qin Zhao, Jinyang Wang, Chengcheng Yin, Peng Zhang et al. "Near-Infrared Light-Sensitive Nano Neuro-Immune Blocker Capsule Relie...
CROSSREF <1%

68 Tao Bao, Yuanlin Deng, Liang Chen, Weijie Sun et al. "Primary tumour resection in non-small cell lung cancer patients with pleural diss...
CROSSREF <1%

69 Weixue Wang, Youzheng Dong, Hong Yi, Lei He, Yuwen Jiang, Lu Long, Zhen Xia, Juxiang Li. "Single-Nucleus RNA Sequencing Reveals...
CROSSREF <1%

70 Xiaocheng Gong, Yunfei Liu, Qian Zhang, Keying Liang, Jinfen Wei, Hongli Du. "LHFPL2 Serves as a Potential Biomarker for M2 Polariz...
CROSSREF <1%

71 Xiaoyu Liu, Xu Xu, Zhuozhuo Wu, Qungang Shan, Ziyin Wang, Zhiyuan Wu, Xiaoyi Ding, Wei Huang, Zhongmin Wang. "Integrated single-...
CROSSREF <1%

Excluded search repositories:

- None

Excluded from document:

- Bibliography

Excluded sources:

- None

Excluded preprints

- None

Manuscript Title: ZFP36L1 Promotes Non-Small Cell Lung Cancer Progression Under Hypoxia by Modulating CXCL9:SPP1 Polarity: A Single-Cell Transcriptomic Study

Abstract

Background: Non-small cell lung cancer (NSCLC) is the predominant lung cancer subtype with high mortality rate. Drug resistance and immune evasion limit its therapeutic outcomes. Specific mechanism of the oncogenic ZFP36L1 in NSCLC remains unclear.

Methods: ScRNA-seq data were analyzed using bioinformatic approaches. Hypoxia-induced alterations in the macrophage CXCL9:SPP1 ratio were assessed by qRT-PCR, WB, immunofluorescence, flow cytometry, and ELISA. Dual-luciferase reporter and ChIP assays were used to validate ZFP36L1-mediated transcriptional regulation of SPP1. In a co-cultivation system of macrophages and NSCLC cells, tumor cell malignancy was evaluated through flow cytometry, CCK-8, Transwell, colony formation, and scratch assays. Patient-derived organoids co-cultured with macrophages were analyzed via H&E, EdU, and CellTiter-Glo assays for pathological changes, proliferation, and viability, as well as qRT-PCR and WB for the expression of apoptosis-related proteins. Finally, through a macrophage-specific ZFP36L1-knockout mouse model, the function of ZFP36L1 in CXCL9:SPP1 polarity and NSCLC progression was validated *in vivo*.

Results: Hypoxia induced an imbalanced macrophage CXCL9:SPP1 ratio, with more pro-tumor $SPP1^+$ macrophages and fewer anti-tumor $CXCL9^+$ macrophages. Upregulation of ZFP36L1 promoted macrophage polarization toward the $SPP1^+$ phenotype, which then bound to CD44 on tumor cells to accelerate NSCLC progression.

Conclusion: Under hypoxia, ZFP36L1 transcriptionally regulates SPP1 to reduce the macrophage

CXCL9:SPP1 ratio, thereby driving NSCLC malignancy.

Keywords: non-small cell lung cancer; macrophage; single-cell transcriptomics; CXCL9:SPP1; ZFP36L1

1 Introduction

Lung cancer constitutes a global health burden, causing nearly 2.5 million new cases and 1.8 million deaths per year. The incidence is substantially higher in men than in women[1]. Non-small cell lung cancer (NSCLC) comprises nearly 85% of all lung cancers[2]. Chemotherapy, targeted therapy, and immunotherapy are now applied in advanced NSCLC treatment[3, 4]. Immune checkpoint blockage using EGFR, ALK and PD-1/PD-L1 inhibitors has improved prognosis substantially[5]. However, drug resistance and tumor microenvironment (TME)-mediated immune evasion continue to be major clinical challenges[6]. Thus, identifying novel biomarkers and developing more effective therapeutic approaches are urgently needed.

The TME, composed of cancer-associated fibroblasts, tumor cells, immune cells, and endothelial cells (ECs), governs tumor initiation, progression, and metastasis[7, 8]. TAMs are predominant immune cells in the TME that contribute to inflammation, immune evasion, and malignant progression[9]. Recent scRNA-seq studies have identified SPP1^{+} TAMs as a distinct macrophage subset linked with NSCLC progression[10-12]. SPP1, a macrophage-derived osteopontin, is involved in cell adhesion, migration, and immune regulation[13]. Reportedly, SPP1 mediates interactions between hepatocellular carcinoma (HCC) cells and macrophages via binding to CD44[14]. The macrophage CXCL9:SPP1 ratio, can indicate either anti-tumor or pro-tumor TAM phenotypes[15]. Regulators of CXCL9:SPP1 ratio in the TME, as well as the specific pathways, deserve further exploration.

ZFP36L1, a member of the ZFP36 gene family, promotes tumor progression in gastric cancer[16]

and glioma[17], and regulates immune responses in diverse immune cells[18]. ZFP36L1 inhibits $CD8^+$ T cell activation to modulate immune responses[19], and enhances IFN-γ-induced PD-L1 expression via super-enhancer-mediated regulation[20]. While it functions in multiple aspects of tumor and immune modulation, its role in macrophages and NSCLC malignancy has not been elucidated.

Through scRNA-seq and cell experiments, our study demonstrates that hypoxia reduces the CXCL9:SPP1 ratio in macrophages and promotes NSCLC progression. ZFP36L1 is upregulated under hypoxia and serves as a key regulator of macrophage CXCL9:SPP1 ratio. Mechanistically, ZFP36L1-induced $SPP1^+$ macrophages enhance NSCLC progression via CD44 binding, providing a promising therapeutic target for NSCLC immunotherapy.

2 Methods

2.1 Data collection

scRNA-seq GSE148071 from the GEO (http://www.ncbi.nlm.nih.gov/geo/) was used, comprising 26 samples from patients with advanced NSCLC (2 NSCLC, 11 LSCC, and 13 LUAD). Additionally, 4 normal lung tissue clinical samples from GSE131907 served as controls.

The RNA sequencing data (TPM) of gene expression and clinical information for TCGA_LUAD were from UCSC Xena (https://gdc.xenahubs.net). Gene expression files, together with the corresponding gene symbol relationships, were converted into Gene ID. In TCGA_LUAD dataset, we grouped the samples using the optimal cutoff. The optimal cutoff for samples sorted into better and worse survival groups was determined by “surv_cutpoint” in the “survminer” R package. Overall survival (OS) and PFI of the samples underwent survival curve analysis.

Gene expression matrix for GSE3141 was downloaded from the GEO, with probes converted into Gene Symbols. In the GSE3141 dataset, we categorized the samples based on the optimal cutoff and conducted survival curve analysis on their OS.

2.2 Single-cell data processing and quality control (QC)

Data from 30 samples were integrated, yielding an initial dataset of 83,417 cells and 18,197 genes. For subsequent analyses, the “Seurat” R package (v4.1.0) was applied. Following QC was applied: (1) Unique gene counts and unique molecular identifiers (UMI) per cell were required to be >100, with no upper limit; (2) Mitochondrial genes per cell were required to be <15%; and (3) Cells with nCount > 50,000 or nFeature > 7,000 were excluded. The final dataset consisted of 80,750 cells and 18,197 genes.

2.3 Cell clustering and annotation

The scaled expression matrix was subjected to PCA with the "RunPCA" function. Cell clusters were constructed from the first 40 principal components (PCs) with "FindNeighbors" and "FindClusters" functions, visualized by UMAP. Cell annotations and subtyping referenced canonical gene markers from the published literature[21] and the CellMarker 2.0 database. The distribution of marker genes was analyzed.

2.4 Transcription factor (TF) analysis

The KnockTF 2.0 database was employed to identify TFs that regulate SPP1 and CXCL9TFs regulating SPP1 and CXCL9. Marker genes for $SPP1^{+}$ and $CXCL9^{+}$ TAMs were obtained by the "FindMarkers" function in Seurat for the intersection with the TFs. The expression of key genes among $SPP1^{+}$, $CXCL9^{+}$, and $SPP1^{-}$/$CXCL9^{-}$ TAMs was assessed through correlation analysis.

2.5 Pathway enrichment analysis

Standard gene sets were all from the MSigDB. Pathway activity was evaluated with the "GSVA" R package. Mean gene expression in each cluster was quantified using the "Seurat" package. Significant enrichment was defined by an Adj. p.val < 0.05. Key pathways were visualized with the "ggplot2" package.

2.6 Cell-cell communication

Seurat objects were normalized and imported into the CellChat toolkit. The "computeCommunProb" function was used to calculate communication probabilities. Significant ligand-receptor interactions within given signaling pathways and signal transduction genes were identified using the "extractEnrichedLR" function to reveal communication numbers and strength among cell populations. The overall communication patterns and signaling networks were visualized and analyzed using built-in CellChat functions.

2.7 Cell cultivation

Human monocytic leukemia THP-1 cells (CL-0233, Procell, China), human NSCLC A549 cells (BNCC337696), human LSCC NCI-H2170 cells (BNCC101664), and mouse Lewis lung carcinoma (LLC) cells (BNCC100069, all from BeNa Culture Collection, China) were used. THP-1 cells were cultivated in RPMI-1640 with 10% FBS (Gibco, USA) and 0.05 mM β-mercaptoethanol (Maokangbio, China), NCI-H2170 cells in 20% FBS-containing RPMI-1640, A549 cells in 10% FBS-containing F-12K medium, and LLC cells in 10% FBS-containing DMEM-H. All media were from Gibco (USA) and contained 1% P-S (Beyotime, China). The condition for cell incubation was 37 °C and 5% CO_2.

THP-1 cells were differentiated to M0 macrophages with 150 nM PMA (Sigma-Aldrich, USA) for 48 h. Hypoxia was mimicked as 1% O_2, 5% CO_2, 94% N_2 using a Tri-gas incubator (Thermo Fisher, USA). After the macrophages were treated under hypoxic conditions for 48 h, the following functional experiments and co-culture experiments were conducted. The supernatant from the resulting M0 macrophages was co-cultivated with tumor cells at a ratio of 1:5 for 48 h.

2.8 Cell transfection

Short hairpin RNAs targeting ZFP36L1 (sh-ZFP36L1 or sh-ZFP36L1#1) and SPP1 (sh-SPP1), ZFP36L1 overexpression plasmid (oe-ZFP36L1), and their controls (sh-NC, oe-NC) were synthesized by GenePharma (China). Transfections into THP-1 cells continued with Lipofectamine™ 3000 (Invitrogen, USA) for 48 h before use.

2.9 qRT-PCR

Total RNA extracted with TRIzol reagent (Invitrogen, USA) were synthesized into cDNA with the HiScript Q RT SuperMix kit (Vazyme, China), which were amplified on an ABI 7500 system with SYBR Green Premix (MCE, USA) (primers in **Table 1**). Gene expression was computed by the $2^{-\Delta\Delta Ct}$,

normalized to β-actin.

2.10 WB

Extracted using RIPA lysis buffer (Beyotime, China), total protein were denatured at 100 °C for 5 min in loading buffer (Beyotime, China), separated by SDS-PAGE, and moved to PVDF membranes (Sigma-Aldrich, USA). After 5% non-fat milk blocking (45 min), membranes were kept with primary antibodies at 4 °Covernight; washed three times with TBST, HRP-conjugated secondary antibodies were added (1 h, room temperature [RT]). With a Tanon-4600 imaging system (Biotanon, China), protein bands were visualized. Antibody information is detailed in **Table 2**.

2.11 Immunofluorescence (IF)

For M0 macrophages, cells on sterile coverslips in 24-well plates were subjected to three PBS washings, 75% ethanol fixation (15 min), and 0.1% Triton X-100 (Beyotime, China) permeabilization (15 min). After 1 h of 3% BSA (Beyotime, China) blocking, incubation with primary antibodies continued at 4 °C overnight; after PBS washings, at RT without lights, fluorophore-conjugated secondary antibodies were introduced for 1 h. After counterstaining with DAPI (Beyotime, China), nuclei were imaged with a fluorescence microscope (Olympus, Japan).

For mouse tissues, sections were immobilized in 4% paraformaldehyde (PFA, Beyotime, China) for 24 h, dehydrated, embedded, and sectioned into 5 μm. After 1 h of 3% BSA blocking 1 h at RT, primary antibodies were applied (4 °C, overnight), and, after three TBST washes, secondary antibodies for 1 h at RT. After DAPI (Beyotime, China) counterstaining, under a fluorescence microscope tissues were recorded (Olympus, Japan). Antibodies for immunofluorescence are detailed in Table 3.

2.12 Flow cytometry

Apoptosis detection used a flow cytometer (BD Biosciences, USA). Cells digested with trypsin without EDTA (Beyotime, China) were centrifuged and rinsed in PBS twice, before being resuspended in binding buffer (500 μL) and stained with Annexin V-FITC and PI (each for 5 μL, 10 min, RT; provided by kit HY-K1073, MCE, USA).

For the detection of SPP1^{+}/CXCL9^{+} TAMs, cells were immobilized with 4% PFA (Beyotime, China) for 1 h at RT. After two washes with pre-cooled permeabilization buffer, cells were kept with anti-CXCL9 (ab290643) and Goat anti-Rabbit IgG Fc (Alexa Fluor® 750) (ab175735, both from Abcam, UK), or anti-SPP1 (6696-RBM3-P0, Thermo Fisher, USA) for 1 h at RT without lights. Permeabilization buffer washing of cells continued twice. Resuspended cells in buffer were assessed on a flow cytometer (BD Biosciences, USA).

2.13 ELISA

IL-7, CXCL10, IL-1β, and CXCL5 levels were tested with commercial ELISA kits. Cell supernatants or serum samples were centrifuged (10 min, 2,000 rpm, 4 °C) before resulting supernatants stored on ice. Then, 100 μL of standards or samples were incubated in a 96-well plate for 2 h at 37 °C before HRP-conjugated antibody (100 μL/well) being applied for 1 h at 37 °C. After rising with 200 μL wash buffer, wells stood for 2 min. Colors were developed with 90 μL of TMB substrate for 30 min without lights. Reaction was stopped by 50 μL of stop solution before measuring optical density (OD; 450 nm) on a microplate reader (Bio-Rad, USA). CXCL10 (Mouse: E-EL-M0021; Human: E-EL-H0050), IL-1β (Mouse: E-EL-M0037; Human: E-EL-H0149), and CXCL5 (Mouse: E-EL-M0471; Human: E-EL-H0046) ELISA kits from Elabscience (China) and IL-7 ELISA kits (Mouse: EMIL7; Human: EHIL7) from Thermo Fisher (USA) were used.

2.14 CCK-8 viability assay

Single-cell suspension was prepared with logarithmic phase co-cultured cells trypsinized (Beyotime, China) at 2,000 cells/mL (100 μL) and incubated in a 96-well plate, for 0, 24, 48, 72, and 96 h, before reaction with CCK-8 reagent (10 μL/well; provided by kit, Beyotime, China) for 1-4 h. OD (450 nm) was tested with a microplate reader (Bio-Rad, USA).

2.15 Colony formation assay

Log-phase cells (100 cells/ well) were put into 12-well plates in 1 mL of culture medium. Every 3 days, Medium was replaced with a new one and after 14 days was removed. After two gentle PBS washes, colonies were immobilized with 4% PFA for 20 min at RT, stained with 0.1% crystal violet solution (15 min; Beyotime, China). After removing excess dye with three PBS rinses, the plates were air-dried at RT. Colonies were imaged with ImageJ (NIH, USA).

2.16 Scratch assay

At an appropriate density, cells were put into 6-well plates. Upon approximately 95% confluence, a sterile 10 μL pipette tip was used to create a straight scratch in the monolayer. Detached cells were removed by three PBS washes. Scratch was imaged at 0 and 48 h using an inverted microscope (Olympus, Japan) and quantified for closure rate by ImageJ (NIH, USA).

2.17 Transwell assays

Cell migration and invasion abilities were tested 24 h post-transfection. After trypsin (Beyotime, China) digestion, cells were resuspended in serum-free medium and adjusted to 1×10^4 cells/mL. To detect migration, the upper and lower chambers of the Transwell system (Corning, USA) were filled with cell suspension (200 μL) and complete medium (500 μL) containing 10% FBS, respectively. To detect invasion, Transwell inserts were pre-coated with Matrigel (BD Biosciences, USA). The incubation lasted for 24 h at 37 °C. With a wet cotton swab, we wiped off non-migrated/invaded cells.

Cells on the lower surface were immobilized with 4% PFA (Beyotime, China) for 20 min, followed by 15 min of 0.5% crystal violet (Beyotime, China) staining. After PBS rinse, cells on the lower membrane surface were recorded with an inverted microscope (Olympus, Japan).

2.18 ChIP assay

Cells were collected and cross-linked with 1% formaldehyde for 10 min. The cross-linking was quenched by 5 min of glycine treatment (MCE, USA; final concentration: 0.125 M) at RT. The cross-linked chromatin was then fragmented by sonication (300-500 bp). Immunoprecipitation was performed by incubating the sonicated samples with Protein A/G magnetic beads (HY-K0202, MCE, USA) pre-adsorbed by specific Rabbit anti-ZFP36L1 (ZFP36L1-101AP, Invitrogen, USA) or IgG isotype control (ab313801, Abcam, UK). Target DNA was finally amplified and detected by qPCR.

2.19 Dual-luciferase reporter assay

ZFP36L1 regulation of the SPP1 promoter was analyzed. Wild-type (WT) and mutant (MUT) promoter fragments of SPP1 were separately cloned into the pGL4Luc-RLuc dual-luciferase vector (Novopro, China). 1 μg of reporter plasmid was co-transfected with oe-ZFP36L1 or oe-NC into 293T cells by Lipofectamine 3000 (Invitrogen, USA). Firefly and Renilla luciferase activities were quantified 48 h later with a commercial kit (Promega, USA) and expressed as Firefly/Renilla ratio.

2.20 Detection of mRNA stability

The processed macrophages were inoculated in 6-well plates and 5 μg/mL of actinomycin D was added. The cells were treated for 0 h, 2 h, 4 h, and 8 h, respectively. Cells were collected for qRT-PCR.

2.21 Patient-derived organoids (PDOs)

Under the approval conferred by the Ethics Committee of Ningbo Medical Center Lihuili

Hospital (Approval number KY2025SL501-01), we gathered tumor and adjacent normal tissues from 40 NSCLC patients. Informed consent of patients was obtained.

Inclusion criteria: age ≥18 years; histopathologically confirmed NSCLC with tissue specimens obtained via fiberoptic bronchoscopy biopsy, pretreatment lung puncture, or surgical resection; clinical stage IIIB-IV or recurrent/metastatic disease; absence of severe comorbidities; at least one measurable lesion according to Response Evaluation Criteria in Solid Tumors; and an Eastern Cooperative Oncology Group scoring of 0-2.

Exclusion criteria: presence of other primary tumors outside the lung; previous adoptive cell therapy, tumor vaccines, or other forms of immunotherapy; previous systemic immunosuppressive therapy; or a history of non-infectious pneumonia or active autoimmune disease requiring steroid treatment.

2.22 PDO cell culture

Fresh NSCLC samples were minced into fragments (≤1 mm^3) and rinsed three times with pre-cooled PBS containing 10% P-S (Beyotime, China). The tissue fragments were digested in pre-cooled digestion buffer, which contains 1% FBS (Gibco, USA), 1.5 mg/mL Collagenase Type II (Thermo Fisher, USA), 500 U/mL Collagenase Type IV (Thermo Fisher, USA), 0.1 mg/mL Dispase II (Beyotime, China), and 10 μM Y-27632 (MCE, USA) at 37 °C with agitation for 45 min. The digest was centrifuged at 200 × g for 5 min. Three PBS washings were run on the pellet. The final single-cell suspension was mixed with Matrigel (BD Biosciences, USA) and seeded into 96-well plates.

Upon Matrigel polymerization, organoids were switched to modified organoid complete medium, i.e., advanced DMEM/F12 (Gibco, USA) containing N-acetyl-L-cysteine (1 mM, Yeasen, China), gastrin (10 nM, Sigma-Aldrich, USA), Noggin (0.1 μg/mL), R-spondin (1 μg/mL), EGF (50 ng/mL),

nicotinamide (10 mM), A83-01 (0.5 μM), and FGF10 (100 ng/mL; MCE, USA), and standard supplements (GlutaMAX, HEPES, N2, B27; Thermo Fisher, USA). Media were refreshed every 3 days; organoids were passaged every 7 days according to growth.

2.23 Organoid-macrophage co-culture

Pre-thawed Matrigel was immobilized with pre-cooled basal medium at 2:3. Using a Transwell system (Corning, USA), 50 μL of the mixture was spread over the upper inserts and incubated at 37 °C for 1 h. After the Matrigel had fully solidified, the organoid cell suspension was seeded onto the inserts. The Transwell system was placed into a 24-well plate pre-seeded with M0 macrophages. They were co-cultured in an appropriate amount of complete medium in a 37 °C, 5% CO_2 incubator for 48 h. Upon the completion of co-culture, Transwell inserts were carefully taken out and organoid cells were collected for use.

2.24 H&E staining

Tissues were immobilized by 4% PFA (Beyotime, China) for 24 h, paraffin-embedded conventionally and sliced into serial 5 μm sections with a microtome. After deparaffinization and rehydration, hematoxylin (Beyotime, China) staining of the section lasted for 5-10 min. The sections were differentiated with 1% acid alcohol for 1-3 s, rinsed in tap water for several seconds to remove excess stain, blued in 1% ammonia water for 10 s, and rinsed in tap water for about 10 min. The sections were counterstained with eosin (Beyotime, China) for 1-2 min. After graded ethanol dehydration, xylene clearing, and neutral balsam mounting, sections were observed with an optical microscope (Olympus, Japan).

2.25 EdU staining

The organoid cell suspension was seeded into 24-well plates containing circular coverslips and

cultured overnight at 37 °C to allow attachment. Then, 1× EdU working solution (CA1170, Solarbio, China) was put into each well and incubated for 2 h at 37 °C. After discarding the media, 4% PFA (Beyotime, China) was applied to fix cells for 15 min at RT, followed by 10 min of 0.3% Triton X-100 (Beyotime, China) permeabilization. After three PBS washes, 100 µL of click reaction mixture (Beyotime, China) was introduced for 30 min RT incubation, protected from the light. After another wash, DAPI (Beyotime, China) was added and incubated for 10 min without lights. Finally, images were captured with a fluorescence microscope (Olympus, Japan).

2.26 CellTiter-Glo assay

Organoid viability was tested with the CellTiter-Glo assay kit (G7570, Promega, USA). Briefly, 5,000 organoid cells were put into 96-well plates. The cultivation lasted for 0, 24, 48, 72, and 96 h. Each well received 80 µL of CellTiter-Glo detection reagent. The plate was shaken at RT for 2 min without lights to mix and then stood for 10 min. Luminescence intensity was tested by a multifunctional microplate reader (Bio-Rad, USA).

2.27 Laboratory animals

C57BL/6J mice with macrophage-specific conditional knockout of ZFP36L1 were produced by GemPharmatech (China). The study involved crossing LysM-Cre mice with Zfp36l1^fl/fl^ mice to obtain macrophage/myeloid cell-specific ZFP36L1 knockout mice (LysM-Cre+/-; Zfp36l1^fl/fl^, hereinafter referred to as ZFP36L1-KO). The control group consisted of littermate LysM-Cre-/-; Zfp36l1^fl/fl^ wild-type mice (hereinafter referred to as WT). Mice (total number = 12) were housed under SPF conditions at the Ningbo Medical Center Lihuili Hospital Laboratory Animal Center, maintained at 22 ± 2 °C and 50 ± 10% humidity with a 12/12-h light/dark cycle. Mice were subcutaneously injected with 1×10^6 of LLC cells (n=6 per group). Tumor growth was monitored on

days 7, 14, 21, and 28. Tumor length (L) and width (W) were tested with calipers to calculate tumor volume (V): $V = L \times W^2/2$. After 4 weeks, mice were euthanized to dissect tumors and record their volume and weight. The animal protocol was completed under the approval conferred by the Ethics Committee of Zhejiang Luoxi Medical Technology Co., Ltd., Hangzhou, China (Approval number LX4825021301).

2.28 Macrophage isolation

Bone marrow cells were utilized and red blood cells were removed with red blood cell lysis buffer (Beyotime, China). To assess cell purity, flow cytometry was conducted, with Mouse anti-F4/80 (11-4801-82, Invitrogen, USA) used. Bone marrow-derived macrophages with >85% F4/80$^+$ purity were used for subsequent experiments.

2.29 Immunohistochemistry (IHC)

Clinical tissue samples and fresh tumor tissues from mice were collected, immobilized with 10% formalin (Maokangbio, China) for 24 h respectively, paraffin-embedded, and sectioned. Deparaffinized and rehydrated sections underwent antigen retrieval in 0.01 M sodium citrate buffer (pH 6.0; Meilunbio, China). After blocking endogenous peroxidase with 3% H_2O_2 (Maokangbio, China) and nonspecific sites with 5% BSA (MCE, USA), sections were kept with primary antibodies (4 °C, overnight) and HRP-conjugated secondary antibodies (1 h) after three PBS washes. Signals were tested with a DAB substrate kit (Sigma-Aldrich, USA). Nuclei were counterstained with hematoxylin (Beyotime, China). After dehydration with ethanol and clearing with xylene, sections were mounted with neutral balsam for photos under a microscope (Olympus, Japan). Antibodies for immunohistochemistry are detailed in Table 4.

2.30 Statistical analysis

Data (mean ± SD) were obtained from independent experiments (minimal count: 3). Statistical analyses and visualization were run in GraphPad Prism 8.0 (GraphPad Software, USA). Between-group difference was tested by Student's *t*-test or one-way ANOVA (p-value < 0.05: statistical significance).

3 Results

3.1 Analysis of macrophage CXCL9:SPP1 ratio in NSCLC based on single-cell datasets

We first analyzed 26 advanced NSCLC samples from the GSE148071 dataset and 4 normal tissue samples from the GSE131907 dataset. QC for the raw scRNA-seq data (nFeature, nCount, and mitochondrial gene percentage) is displayed in violin plots **(Supplementary Fig. 1A)**. Point density plots illustrate the correlation between gene expression features and mitochondrial content before filtering **(Supplementary Fig. 1B)**. Gene expression characteristics of retained cells after QC are shown in **Supplementary Fig. 1C-D**. Following data normalization, 3,000 highly variable genes were identified **(Supplementary Fig. 1E)**. PCA effectively reduced data dimensionality and identified key PCs **(Supplementary Fig. 1F)**. Using the top 40 PCs, cells were partitioned into 28 clusters through the "FindNeighbors" and "FindClusters" functions. The proportion distribution of each cell type and the characteristics of the sample sources are shown in **Fig. 1A-B**. Based on marker genes from published literature[21] and the CellMarker 2.0 database, we annotated 13 major cell types: T cells, ECs, monocytes, dendritic cells, B cells, mast cells, smooth muscle cells, NK cells, macrophages, epithelial cells, alveolar cells, fibroblasts, and tumor cells **(Fig. 1C-D)**. We also visualized the proportions of each cell type (**Fig. 1E-F**). After extracting the above macrophages and conducting re-dimensionality reduction and clustering analysis, two subgroups were finally identified: normal macrophages and TAM (**Fig. 1G**) (**Supplementary Fig. 1G-H**). The proportions of normal macrophages and TAM in distinctive samples are shown in **Fig. 1H**. Then, the TAM subgroups were further clustered, and three subgroups were identified: SPP1+TAM (high expression of SPP1), CXCL9+TAM (high expression of CXCL9), and CPM+TAM (SPP1/CXCL9 double-negative), with SPP1+TAM accounting for the majority (**Fig. 1I**). These findings indicate that TAM demonstrates significant

heterogeneity in the NSCLC microenvironment.

3.2 Hypoxia decreases the macrophage CXCL9:SPP1 ratio and promotes NSCLC progression

The generation of $SPP1^+$ TAMs is closely associated with a hypoxic microenvironment[15]. The impact of macrophage CXCL9:SPP1 ratio on NSCLC progression was investigated. First, M0 macrophages were differentiated from THP-1 cells using PMA and cultivated in either normoxia or hypoxia. qRT-PCR and WB revealed that hypoxia suppressed CXCL9 expression while increasing both SPP1 mRNA and protein levels **(Fig. 2A-B)**, which was confirmed by IF staining **(Fig. 2C)**. Flow cytometry revealed a decreased proportion of $CXCL9^+$/$SPP1^+$ macrophages in the hypoxic group **(Fig. 2D)**, confirming that hypoxia induced a decrease in the CXCL9:SPP1 ratio. MMP9 and MMP12 are reported as markers of $SPP1^+$ TAMs[22]. WB results demonstrated that hypoxia significantly enhanced MMP9 and MMP12 protein expression **(Fig. 2E)**, which is in line with the induction of $SPP1^+$ TAMs. CXCL9, CXCL10 and IL-7 are enriched in the high CXCL9:SPP1 ratio subset, whereas CXCL5 and IL-1β dominate the low-ratio population[15]. In hypoxic macrophages, ELISA revealed decreased production of IL-7 and CXCL10 while increasing IL-1β and CXCL5 levels **(Fig. 2F)**. To probe into the effect of hypoxia-induced macrophage polarization on tumor cell behavior, we co-cultivated normoxia/hypoxia-treated macrophages with A549 or NCI-H2170 cells independently. In functional assays, hypoxic macrophages enhanced tumor cell proliferation, migration, and invasion, while inhibiting apoptosis **(Fig. 2G-K)**. In conclusion, hypoxia promotes NSCLC malignant progression by reducing the macrophage CXCL9:SPP1 ratio.

3.3 ZFP36L1 is a key gene regulating the macrophage CXCL9:SPP1 ratio

To explore factors influencing the polarization balance between $SPP1^+$ and $CXCL9^+$ TAMs, we

queried the KnockTF 2.0 database for TFs of SPP1 or CXCL9. Using the "FindMarkers" function, we identified marker genes for SPP1$^+$ TAMs and CXCL9$^+$ TAMs and intersected them with the TFs, yielding eight genes related to SPP1$^+$ or CXCL9$^+$ TAM polarization. Correlation analysis confirmed a key overall linkage between the gene set and the SPP1/CXCL9 gene **(Fig. 3A)**. We selected the significantly differentially expressed CITED2 and ZFP36L1 for further analysis **(Fig. 3B)**. Gene expression patterns in the SPP1$^+$, CXCL9$^+$, and CPM$^+$ TAM subsets are shown in **Fig. 3C**. CPM$^+$ TAMs expressed immune-related genes (CPM, RARRES1, PLTP), SPP1$^+$ TAMs upregulated genes associated with SPP1 induction, (CITED2, ZFP36L1, MMP9, MMP12), while CXCL9$^+$ TAMs were characterized by chemokine genes such as CXCL10 and CCL5. ZFP36L1, known to suppress inflammatory mediators and exert regulatory effects under hypoxia[18, 23], showed substantial expression divergence between SPP1$^+$ and CXCL9$^+$ TAM subsets **(Fig. 3B)**. ZFP36L1 was thus selected for further investigation. To investigate the correlation between ZFP36L1 and CXCL9:SPP1 ratio and the OS and PFI of patients, we grouped the samples using the optimal cutoff and conducted survival curve analysis for OS and PFI. **Supplementary Fig. 2A-D** exhibit the Kaplan-Meier curves of OS and PFI for ZFP36L1 and CXCL9:SPP1 ratio. Patients with high expression of ZFP36L1 had a poorer prognosis, while those with a high CXCL9:SPP1 ratio had a better prognosis. In the GSE3141 dataset, we used the optimal cutoff for grouping and conducted survival curve analysis for OS. **Supplementary Fig. 2E-F** shows the OS curves of ZFP36L1 and CXCL9:SPP1 ratio, which are consistent with the results from TCGA database. Patients with high expression of ZFP36L1 had a poorer prognosis, while patients with high CXCL9:SPP1 ratio had a better prognosis. To explore the expression of ZFP36L1 and SPP1 in NSCLC tissues, we used IHC to divide the tissue samples into high and low expression groups of ZFP36L1. The IHC results also showed that compared to the

ZFP36L1 low-expression group, the expression level of SPP1 in the ZFP36L1 high-expression group was increased (**Fig. 3D**). Immunofluorescence staining experiments confirmed that in the ZFP36L1 high-expression group, the co-localization signal of macrophage marker CD11b with ZFP36L1 significantly increased (**Fig. 3E**).

M0 macrophages were cultured under normoxia or hypoxia. qRT-PCR and WB showed that hypoxia upregulated ZFP36L1 in M0 macrophages **(Fig. 3F-G)**. We knocked down ZFP36L1 in THP-1 cells and induced M0 macrophages, and further exposed them to a hypoxic environment. WB examined ZFP36L1, SPP1, and CXCL9 expression. ZFP36L1 knockdown downregulated SPP1 and upregulated CXCL9 **(Fig. 3H)**, which was validated by IF staining **(Fig. 3I)**. Flow cytometry showed that ZFP36L1 knockdown increased the $CXCL9^{+}/SPP1^{+}$ TAM ratio under hypoxia **(Fig. 3J)**. ZFP36L1 knockdown suppressed MMP9 and MMP12 protein expression **(Fig. 3K)** and increased IL-7 and CXCL10 levels while decreasing IL-1β and CXCL5 levels **(Fig. 3L)**. We next investigated whether ZFP36L1 transcriptionally regulates SPP1. ChIP assays confirmed ZFP36L1 binding to the SPP1 promoter region **(Fig. 3M)**. Luciferase reporter assays showed that ZFP36L1 overexpression increased relative luciferase activity for the SPP1-WT promoter but not the SPP1-MUT promoter **(Fig. 3N)**. We also conducted an actinomycin D experiment to assess the post-transcriptional stability of SPP1 mRNA. Silencing ZFP36L1 did not affect the stability of SPP1 mRNA in macrophages (**Fig. 3O**). In conclusion, ZFP36L1 critically regulates the macrophage CXCL9:SPP1 ratio and promotes NSCLC progression under hypoxia by modulating SPP1 and CXCL9 expression.

3.4 ZFP36L1-mediated regulation of macrophage CXCL9:SPP1 ratio influences NSCLC progression

To probe into the impact of ZFP36L1 on macrophage CXCL9:SPP1 ratio and NSCLC progression,

THP-1 cells were divided into oe-ZFP36L1+sh-NC, oe-NC+sh-SPP1, oe-ZFP36L1+sh-SPP1, and oe-NC+sh-NC groups. qRT-PCR and WB confirmed that ZFP36L1 overexpression significantly upregulated SPP1, which were partially counteracted by SPP1 knockdown **(Fig. 4A-B)**. Under hypoxia, the promotive effect of ZFP36L1 overexpression on MMP9 and MMP12 expression was evidently inhibited by SPP1 knockdown **(Fig. 4C)**. ELISA revealed that ZFP36L1 overexpression inhibited IL-7 and CXCL10 secretion while promoting IL-1β and CXCL5 levels; SPP1 knockdown produced opposite effects. ZFP36L1 overexpression could also reverse the effects of SPP1 knockdown on IL-7, CXCL10, IL-1β and CXCL5 **(Fig. 4D)**. Hypoxic M0 macrophages from the four groups were co-cultured with A549 or NCI-H2170 cells. In the co-culture system, ZFP36L1 overexpression promoted tumor cell proliferation, migration and invasion, and inhibited apoptosis; SPP1 knockdown induced the opposite results. ZFP36L1 overexpression reversed the effects induced by SPP1 knockdown **(Fig. 4E-I)**.

Furthermore, we treated the M0 macrophages in the co-culture system with SPP1 monoclonal antibody (HY-P80771, MCE, USA), and constructed the following groups: oe-NC+IgG, oe-ZFP36L1+IgG, oe-NC+SPP1a, oe-ZFP36L1+SPP1a. Simultaneously, hypoxia treatment was carried out. The treated M0 macrophages were then co-cultured with A549 and NCI-H2170 cells. ZFP36L1 overexpression enhanced tumor cells' growth, migration and invasion abilities in the co-culture system, while repressing cell apoptosis. However, the opposite results were observed after SPP1 monoclonal antibody treatment. These results indicate that the SPP1 monoclonal antibody reverses the tumor-promoting effect caused by ZFP36L1 overexpression (**Supplementary Fig. 3A-E**).

To validate the ZFP36L1-SPP1 axis in NSCLC biologically, we co-cultured the differentially

treated macrophages with NSCLC PDOs under hypoxic conditions. H&E staining revealed that ZFP36L1 overexpression induced extensive inflammatory infiltration, disrupted tissue architecture, and cellular atrophy compared with the control group; SPP1 knockdown attenuated these pathological alterations. However, when ZFP36L1 was overexpressed in SPP1-knockdown M0 macrophages, these pathological changes were reversed **(Fig. 5A)**. Moreover, EdU staining and CellTiter-Glo assays demonstrated that SPP1 knockdown significantly suppressed the pro-proliferative and viability-enhancing effects induced by ZFP36L1 overexpression **(Fig. 5B-C)**. qRT-PCR and WB analyses of apoptosis-related proteins showed that ZFP36L1 overexpression upregulated the anti-apoptotic Bcl-2 and downregulated the pro-apoptotic Caspase-3; SPP1 knockdown resulted in the opposite outcome, which was reversed by overexpressing ZFP36L1 **(Fig. 5D-E)**. In conclusion, ZFP36L1 mediates macrophage CXCL9:SPP1 ratio by regulating SPP1 expression, thereby promoting NSCLC progression.

3.5 ZFP36L1-driven SPP1^{+} TAM targets tumor cell surface CD44 to accelerate NSCLC progression

GSVA (KEGG) pathway enrichment analysis revealed that SPP1^{+} TAMs significantly activated multiple pro-tumorigenic pathways, notably the folate biosynthesis pathway associated with M2 polarization and the sulfur metabolism pathway linked to anti-inflammatory responses **(Fig. 6A)**. Pseudotime analysis indicated that under hypoxia, ZFP36L1 and CITED2 were upregulated in SPP1^{+} TAMs, promoting macrophage polarization towards the SPP1^{+} phenotype **(Fig. 6B)**. SPP1 upregulation coincided with significant increases in MMP9 and MMP12, key effector molecules in NSCLC invasion and metastasis; CXCL9 expression showed a rise and fall trend in the pseudotime progression **(Fig. 6C)**. Expression patterns of genes collectively modulate NSCLC development.

Differentiated cell-cell communication patterns between the three TAM subsets (CPM$^+$, SPP1$^+$, CXCL9$^+$) and 12 other cell types were revealed by CellChat **(Fig. 6D)**. SPP1$^+$ TAM, as a signal receiver, engaged in cell-cell communication through the MIF-(CD74+CXCR4) ligand-receptor pair **(Fig. 6E)**; as a signal sender, it primarily interacted with various integrin receptors, including CD44, ITGAV+ITGB1, and ITGA5+ITGB1 **(Fig. 6F)**.

To investigate SPP1 and CD44 expression in NSCLC tissues, we sub-grouped the tissue samples. Immunofluorescence staining confirmed that in the SPP1 high-expression group, the levels of SPP1 and CD44 significantly increased (**Fig. 7A**). The IHC demonstrated that compared to the SPP1 low-expression group, CD44 was higher in the SPP1 high-expression group (**Fig. 7B**). Given that SPP1$^+$ TAM can secrete SPP1 that binds to CD44, a well-characterized surface marker on tumor cells[24, 25], we hypothesized that SPP1$^+$ TAM promotes NSCLC through CD44. Using a CD44 monoclonal antibody (HY-P80062, MCE, USA) in the co-culture system of M0 macrophage and A549 or NCI-H2170 cells under hypoxia, we evaluated the malignancy of tumor cells in the following groups: oe-NC+IgG, oe-ZFP36L1+IgG, oe-NC+CD44a, and oe-ZFP36L1+CD44a, through CCK-8, colony formation, Transwell and scratch assays, and flow cytometry. Compared to the control, anti-CD44 treatment alone significantly attenuated ZFP36L1 overexpression-induced enhancement of proliferation, migration, invasion, and anti-apoptosis **(Fig. 7C-G)**. In conclusion, ZFP36L1 accelerates NSCLC progression by promoting the SPP1$^+$ phenotype and increasing SPP1 to bind to CD44 on tumor cells.

3.6 *In vivo*, ZFP36L1 knockout in macrophages inhibits NSCLC progression by balancing CXCL9:SPP1 ratio

In vitro, ZFP36L1 promotes NSCLC progression by modulating macrophage CXCL9:SPP1 ratio.

To test this *in vivo*, we generated a mouse model with conditional knockout of ZFP36L1 in macrophages. Tumor growth was monitored and macrophages were isolated from mouse tissues. qRT-PCR and WB confirmed the absence of ZFP36L1 expression in the ZFP36L1-KO group **(Fig. 8A)**. ZFP36L1 knockout repressed tumor volume and weight, compared to the wild-type (WT) **(Fig. 8B-D)**. H&E staining revealed distinct pathological changes in mouse tumor tissues. WT tissues showed solid sheets of highly differentiated, spindle- or oval-shaped tumor cells, with evident localized nuclear division (black arrows) and large, well-demarcated necrotic areas containing abundant proteinaceous fibrin from necrotic cells (red arrow). ZFP36L1-KO tissues showed widespread necrosis with clear boundaries and abundant proteinaceous fibrin (red arrow); the remaining tumor parenchyma was peripherally concentrated, with unclear, occasional mitotic figures (black arrows) and mild degenerative necrosis (yellow arrow); the stroma showed no identifiable fibroblastic septa **(Fig. 8E)**. IHC analysis showed that ZFP36L1 knockout significantly downregulated SPP1, CD44, and Ki67 protein expression and upregulated CXCL9 **(Fig. 8F)**. IF confirmed increased co-localization of the macrophage marker F4/80 with CXCL9 and decreased co-localization with SPP1 in the ZFP36L1-KO group **(Fig. 8G)**. ELISA indicated that ZFP36L1 deficiency significantly increased IL-7 and CXCL10 levels and decreased IL-1β and CXCL5 in the TME **(Fig. 8H)**. In conclusion, ZFP36L1 knockout in macrophages suppresses NSCLC progression *in vivo* by modulating CXCL9:SPP1 ratio balance.

4 Discussion

NSCLC is a malignancy with high incidence and mortality, whose significant tumor heterogeneity frequently results in suboptimal treatment outcomes[26]. Exploring novel molecular biomarkers is therefore of great importance. In recent years, scRNA-seq analysis has attracted attention in various malignant tumors, such as pancreatic ductal adenocarcinoma[27], nasopharyngeal carcinoma[28], and NSCLC[21], providing new insights into how immune cells within the TME influence malignant progression. The scRNA-seq was used to explore the function of the macrophage CXCL9:SPP1 ratio in NSCLC. Building on this, we combined cell and animal experiments to demonstrate that ZFP36L1 promotes the secretion of SPP1 by increasing the proportion of $SPP1^{+}$ TAMs, which in turn binds to CD44 on tumor cells, ultimately driving malignant progression. These findings define ZFP36L1's role in NSCLC and establish it as a therapeutic target, offering a theoretical basis for macrophage polarization-based intervention strategies.

The functional heterogeneity of the TME is determined by its TAM polarization. Pro-inflammatory M1 TAMs exert anti-tumor effects by releasing pro-inflammatory factors and reactive oxygen species. Anti-inflammatory M2 TAMs, on the other hand, drive tumor progression by releasing growth factors and immunosuppressive cytokines[29]. A distinct SPP1-high TAM subset ($SPP1^{+}$ TAMs) tightly linked to tumor necrosis was identified[30]. $SPP1^{+}$ TAMs are considered to be pro-tumorigenic in various cancers, such as colorectal cancer[30], HCC[22], and NSCLC, and are linked to poor prognosis. Recent scRNA-seq analyses have revealed a mutual exclusivity in CXCL9 and SPP1 expression in macrophages, with their ratio determining whether macrophages exhibit pro- or anti-tumor phenotypes[15]. Gu *et al.*[31] identified the CXCL9:SPP1 ratio as a prognostic predictor for HCC. Consistently, our scRNA-seq analysis found that $SPP1^{+}$ TAMs constitute the largest proportion

among macrophage subpopulations and are associated with NSCLC progression. This suggests that modulating the CXCL9:SPP1 ratio in TAMs could represent a novel therapeutic direction, although the underlying mechanisms require further exploration.

Hypoxia promotes the generation of $SPP1^+$ TAMs to promote cancer malignant progression[32]. Hypoxia is a key factor in the TME that drives malignant progression by influencing metabolic reprogramming, angiogenesis, and tumor immunity[33, 34]. In NSCLC, hypoxia can significantly promote tumor cell migration and invasion[35]. Additionally, hypoxia participates in tumorigenesis and development by regulating the expression of specific CXC chemokines in the TME[36]. For instance, HIF-1α transcriptionally suppresses chemokines (CXCL9, CXCL10, and CXCL11), and reduces $CD8^+$ T cell infiltration in the TME of colorectal cancer, thereby promoting tumor immune evasion and malignant progression[37]. Similarly, in our study, hypoxia downregulated CXCL9 and CXCL10 in macrophages while upregulating CXCL5 and SPP1, leading to a significant decrease in the CXCL9:SPP1 ratio in macrophages. Co-culture of hypoxic macrophages with NSCLC cells promoted malignant cancer phenotypes. This indicates that hypoxia drives a decrease in the macrophage CXCL9:SPP1 ratio, inducing NSCLC progression. Therefore, identifying key factors in the TME that drive macrophage polarization towards the $SPP1^+$ phenotype is crucial.

We identified ZFP36L1 as a key target gene influencing CXCL9:SPP1 ratio through scRNA-seq. ZFP36L1 downregulates inflammatory factors and exerts regulatory functions in hypoxic environments[18, 23]. Notably, ZFP36L1 overexpression is documented in gastric cancer tissues and closely correlates with worse prognosis, suggesting its oncogenic role[16]. However, the specific impact of ZFP36L1 on NSCLC progression under hypoxia had not been clearly established until our research. By co-culturing hypoxic macrophages with A549 or NCI-H2170 cells, we demonstrated that ZFP36L1

overexpression promoted tumor cell growth, migration, and invasion, and inhibited apoptosis, confirming its tumor-promoting effects. Two studies on lung cancer have reported the tumor suppressor role of ZFP36L1. The first study found that ZFP36L1 inhibited LUAD cells' proliferation, survival, and cycle progression[38]. The second study showed that the R9-ZFP36L1 fusion protein could significantly inhibit tumor growth in nude mice and lower the level of various angiogenic and inflammatory cytokines[39]. The results of this study are contrary to previous studies. We hypothesize the reasons as follows. Previous studies were mostly conducted under normoxia conditions, while this study simulated the hypoxic characteristics of the tumor microenvironment (1% O_2). The hypoxic environment may change the metabolic state and gene expression profile of macrophages, reshape the target recognition ability of ZFP36L1, and lead to the promotion of the malignant progression of tumor cells. Cell-cell communication analysis revealed the binding of SPP1 and CD44 on the tumor cell surface when $SPP1^+$ TAMs act as the sender. CD44 is a non-kinase cell surface transmembrane glycoprotein, whose upregulation can promote malignant tumor progression[25]. In the co-cultivation system of macrophages with NSCLC cells under hypoxia, we revealed that adding the CD44 monoclonal antibody inhibited the malignant phenotype of the tumor cells. Notably, knocking down ZFP36L1 reversed these effects. In conclusion, ZFP36L1 promotes macrophage polarization towards the $SPP1^+$ phenotype, accelerating NSCLC progression by binding to CD44 on tumor cells. This potentially offers a new research direction for overcoming the immunosuppressive TME in NSCLC. The preliminary ChIP and dual luciferase experiments in this study are not sufficient to distinguish direct transcriptional activation from indirect regulatory mechanisms, and knocking down ZFP36L1 does not affect the stability of SPP1 mRNA in macrophages. Based on these findings, we propose two possible regulatory hypotheses: (1) ZFP36L1 may degrade the mRNA of a repressive

transcription factor, thereby relieving its inhibitory effect on the SPP1 promoter and indirectly upregulating SPP1 expression; (2) ZFP36L1 may interact with non-coding RNAs (such as lncRNAs) or transcription machinery components and be recruited to the SPP1 promoter region to participate in regulation. However, the specific molecular mechanism between ZFP36L1 and SPP1 still needs to be further elucidated. In subsequent studies, we plan to use RNA immunoprecipitation sequencing technology to identify the genome-wide RNA binding targets of ZFP36L1 in hypoxic macrophages, and use CRISPR-Cas9 technology to edit the potential ZFP36L1 binding motifs in the SPP1 promoter region for function verification.

In summary, ZFP36L1 is a key regulator of macrophage CXCL9:SPP1 ratio in NSCLC progression. Through cellular experiments and animal model validation, we confirmed that hypoxia-induced upregulation of ZFP36L1 promotes NSCLC progression by decreasing the CXCL9:SPP1 ratio in macrophages **(Fig. 9)**. However, certain limitations should be acknowledged. First, bioinformatic analysis had a limited sample size, with scRNA-seq data derived from only 4 control samples and 26 NSCLC samples. Additionally, the function of the SPP1-CD44 interaction *in vivo* has not been validated. Given the core innovation points of the current research, we did not conduct a systematic and comprehensive verification of the upstream hypoxia regulatory mechanism of ZFP36L1 and the various downstream signaling pathways of SPP1-CD44. Sample size should be expanded in future research tovalidate the SPP1-CD44 axis in NSCLC progression *in vivo*. We plan to systematically identify the upstream hypoxia mechanism of ZFP36L1, the specific downstream pathways activated by SPP1-CD44, and their contribution in NSCLC through phosphorylation protein chips, RNA-seq, and functional rescue experiments.

References

1. Bray, F.; Laversanne, M.; Sung, H.; Ferlay, J.; Siegel, R. L.; Soerjomataram, I.; Jemal, A., Global cancer statistics 2022: GLOBOCAN estimates of incidence and mortality worldwide for 36 cancers in 185 countries. *CA Cancer J Clin* **2024,** *74* (3), 229-263.

2. Bravaccini, S.; Bronte, G.; Ulivi, P., TMB in NSCLC: A Broken Dream? *Int J Mol Sci* **2021,** *22* (12).

3. Miao, D.; Zhao, J.; Han, Y.; Zhou, J.; Li, X.; Zhang, T.; Li, W.; Xia, Y., Management of locally advanced non-small cell lung cancer: State of the art and future directions. *Cancer Commun (Lond)* **2024,** *44* (1), 23-46.

4. Jeon, H.; Wang, S.; Song, J.; Gill, H.; Cheng, H., Update 2025: Management of Non-Small-Cell Lung Cancer. *Lung* **2025,** *203* (1), 53.

5. Gargalionis, A. N.; Papavassiliou, K. A.; Papavassiliou, A. G., Immune Checkpoint Inhibitors in Non-Small Cell Lung Cancer (NSCLC) Treatment: Quo Vadis? *Int J Mol Sci* **2024,** *25* (12).

6. Chen, J.; Alduais, Y.; Chen, B., Therapeutic and Systemic Adverse Events of Immune Checkpoint Inhibitors Targeting the PD-1/PD-L1 axis for Clinical Management of NSCLC. *Cell Transplant* **2021,** *30*, 9636897211041587.

7. Gao, J.; Liang, Y.; Wang, L., Shaping Polarization Of Tumor-Associated Macrophages In Cancer Immunotherapy. *Front Immunol* **2022,** *13*, 888713.

8. Basak, U.; Sarkar, T.; Mukherjee, S.; Chakraborty, S.; Dutta, A.; Dutta, S.; Nayak, D.; Kaushik, S.; Das, T.; Sa, G., Tumor-associated macrophages: an effective player of the tumor microenvironment. *Front Immunol* **2023,** *14*, 1295257.

9. Xiao, Y.; Yu, D., Tumor microenvironment as a therapeutic target in cancer. *Pharmacol Ther* **2021,** *221*, 107753.

10. Qi, J.; Sun, H.; Zhang, Y.; Wang, Z.; Xun, Z.; Li, Z.; Ding, X.; Bao, R.; Hong, L.; Jia, W.; Fang, F.; Liu, H.; Chen, L.; Zhong, J.; Zou, D.; Liu, L.; Han, L.; Ginhoux, F.; Liu, Y.; Ye, Y.; Su, B., Single-cell and spatial analysis reveal interaction of FAP(+) fibroblasts and SPP1(+) macrophages in colorectal cancer. *Nat Commun* **2022,** *13* (1), 1742.

11. Hu, J.; Zhang, L.; Xia, H.; Yan, Y.; Zhu, X.; Sun, F.; Sun, L.; Li, S.; Li, D.; Wang, J.; Han, Y.; Zhang, J.; Bian, D.; Yu, H.; Chen, Y.; Fan, P.; Ma, Q.; Jiang, G.; Wang, C.; Zhang, P., Tumor microenvironment remodeling after neoadjuvant immunotherapy in non-small cell lung cancer revealed by single-cell RNA sequencing. *Genome Med* **2023,** *15* (1), 14.

12. Hao, B.; Zhang, Z.; Lu, Z.; Xiong, J.; Fan, T.; Song, C.; He, R.; Zhang, L.; Pan, S.; Li, D.; Meng, H.; Lin, W.; Luo, B.; Yang, J.; Li, N.; Geng, Q., Single-cell RNA sequencing analysis revealed cellular and molecular immune profiles in lung squamous cell carcinoma. *Transl Oncol* **2023,** *27*, 101568.

13. Zhao, Y.; Huang, Z.; Gao, L.; Ma, H.; Chang, R., Osteopontin/SPP1: a potential mediator between immune cells and vascular calcification. *Front Immunol* **2024,** *15*, 1395596.

14. Liu, L.; Zhang, R.; Deng, J.; Dai, X.; Zhu, X.; Fu, Q.; Zhang, H.; Tong, Z.; Zhao, P.; Fang, W.; Zheng, Y.; Bao, X., Construction of TME and Identification of crosstalk between malignant cells and macrophages by SPP1 in hepatocellular carcinoma. *Cancer Immunol Immunother* **2022,** *71* (1), 121-136.

15. Bill, R.; Wirapati, P.; Messemaker, M.; Roh, W.; Zitti, B.; Duval, F.; Kiss, M.; Park, J. C.; Saal, T. M.; Hoelzl, J.; Tarussio, D.; Benedetti, F.; Tissot, S.; Kandalaft, L.; Varrone, M.; Ciriello, G.; McKee, T. A.; Monnier, Y.; Mermod, M.; Blaum, E. M.; Gushterova, I.; Gonye, A. L. K.; Hacohen, N.; Getz, G.; Mempel, T. R.; Klein, A. M.; Weissleder, R.; Faquin, W. C.; Sadow, P. M.; Lin, D.; Pai, S. I.; Sade-Feldman, M.; Pittet, M. J., CXCL9:SPP1 macrophage polarity identifies a network of cellular programs that control human cancers. *Science* **2023,** *381* (6657), 515-524.

16. Ding, K.; Zhang, F.; Qi, G.; Lin, M.; Chen, M.; Chen, Y.; Zheng, J.; Zhou, F., ZFP36L1 Promotes Gastric Cancer Progression via Regulating JNK and p38 MAPK Signaling Pathways. *Recent Pat Anticancer Drug Discov* **2023,** *18* (1), 80-91.
17. Weng, Q.; Wang, J.; Wang, J.; He, D.; Cheng, Z.; Zhang, F.; Verma, R.; Xu, L.; Dong, X.; Liao, Y.; He, X.; Potter, A.; Zhang, L.; Zhao, C.; Xin, M.; Zhou, Q.; Aronow, B. J.; Blackshear, P. J.; Rich, J. N.; He, Q.; Zhou, W.; Suva, M. L.; Waclaw, R. R.; Potter, S. S.; Yu, G.; Lu, Q. R., Single-Cell Transcriptomics Uncovers Glial Progenitor Diversity and Cell Fate Determinants during Development and Gliomagenesis. *Cell Stem Cell* **2019,** *24* (5), 707-723 e8.
18. Makita, S.; Takatori, H.; Nakajima, H., Post-Transcriptional Regulation of Immune Responses and Inflammatory Diseases by RNA-Binding ZFP36 Family Proteins. *Front Immunol* **2021,** *12*, 711633.
19. Petkau, G.; Mitchell, T. J.; Chakraborty, K.; Bell, S. E.; V, D. A.; Matheson, L.; Turner, D. J.; Saveliev, A.; Gizlenci, O.; Salerno, F.; Katsikis, P. D.; Turner, M., The timing of differentiation and potency of CD8 effector function is set by RNA binding proteins. *Nat Commun* **2022,** *13* (1), 2274.
20. Wei, X.; Liu, J.; Cheng, J.; Cai, W.; Xie, W.; Wang, K.; Lin, L.; Hou, J.; Cai, J.; Zhuo, H., Super-enhancer-driven ZFP36L1 promotes PD-L1 expression in infiltrative gastric cancer. *Elife* **2024,** *13*.
21. Wu, F.; Fan, J.; He, Y.; Xiong, A.; Yu, J.; Li, Y.; Zhang, Y.; Zhao, W.; Zhou, F.; Li, W.; Zhang, J.; Zhang, X.; Qiao, M.; Gao, G.; Chen, S.; Chen, X.; Li, X.; Hou, L.; Wu, C.; Su, C.; Ren, S.; Odenthal, M.; Buettner, R.; Fang, N.; Zhou, C., Single-cell profiling of tumor heterogeneity and the microenvironment in advanced non-small cell lung cancer. *Nat Commun* **2021,** *12* (1), 2540.
22. Fan, G.; Xie, T.; Li, L.; Tang, L.; Han, X.; Shi, Y., Single-cell and spatial analyses revealed the co-location of cancer stem cells and SPP1+ macrophage in hypoxic region that determines the poor prognosis in hepatocellular carcinoma. *NPJ Precis Oncol* **2024,** *8* (1), 75.
23. Loh, X. Y.; Sun, Q. Y.; Ding, L. W.; Mayakonda, A.; Venkatachalam, N.; Yeo, M. S.; Silva, T. C.; Xiao, J. F.; Doan, N. B.; Said, J. W.; Ran, X. B.; Zhou, S. Q.; Dakle, P.; Shyamsunder, P.; Koh, A. P.; Huang, R. Y.; Berman, B. P.; Tan, S. Y.; Yang, H.; Lin, D. C.; Koeffler, H. P., RNA-Binding Protein ZFP36L1 Suppresses Hypoxia and Cell-Cycle Signaling. *Cancer Res* **2020,** *80* (2), 219-233.
24. Fu, M.; Shu, S.; Peng, Z.; Liu, X.; Chen, X.; Zeng, Z.; Yang, Y.; Cui, H.; Zhao, R.; Wang, X.; Du, L.; Wu, M.; Feng, W.; Song, J., Single-Cell RNA Sequencing of Coronary Perivascular Adipose Tissue From End-Stage Heart Failure Patients Identifies SPP1(+) Macrophage Subpopulation as a Target for Alleviating Fibrosis. *Arterioscler Thromb Vasc Biol* **2023,** *43* (11), 2143-2164.
25. Hassn Mesrati, M.; Syafruddin, S. E.; Mohtar, M. A.; Syahir, A., CD44: A Multifunctional Mediator of Cancer Progression. *Biomolecules* **2021,** *11* (12).
26. Yang, Q.; Zhang, H.; Wei, T.; Lin, A.; Sun, Y.; Luo, P.; Zhang, J., Single-Cell RNA Sequencing Reveals the Heterogeneity of Tumor-Associated Macrophage in Non-Small Cell Lung Cancer and Differences Between Sexes. *Front Immunol* **2021,** *12*, 756722.
27. Chen, K.; Wang, Q.; Li, M.; Guo, H.; Liu, W.; Wang, F.; Tian, X.; Yang, Y., Single-cell RNA-seq reveals dynamic change in tumor microenvironment during pancreatic ductal adenocarcinoma malignant progression. *EBioMedicine* **2021,** *66*, 103315.
28. Gong, L.; Kwong, D. L.; Dai, W.; Wu, P.; Li, S.; Yan, Q.; Zhang, Y.; Zhang, B.; Fang, X.; Liu, L.; Luo, M.; Liu, B.; Chow, L. K.; Chen, Q.; Huang, J.; Lee, V. H.; Lam, K. O.; Lo, A. W.; Chen, Z.; Wang, Y.; Lee, A. W.; Guan, X. Y., Comprehensive single-cell sequencing reveals the stromal dynamics and tumor-specific characteristics in the microenvironment of nasopharyngeal carcinoma. *Nat Commun* **2021,** *12* (1), 1540.
29. Boutilier, A. J.; Elsawa, S. F., Macrophage Polarization States in the Tumor Microenvironment. *Int J Mol Sci* **2021,** *22* (13).

30. Matusiak, M.; Hickey, J. W.; van, I. D. G. P.; Lu, G.; Kidzinski, L.; Zhu, S.; Colburg, D. R. C.; Luca, B.; Phillips, D. J.; Brubaker, S. W.; Charville, G. W.; Shen, J.; Loh, K. M.; Okwan-Duodu, D. K.; Nolan, G. P.; Newman, A. M.; West, R. B.; van de Rijn, M., Spatially Segregated Macrophage Populations Predict Distinct Outcomes in Colon Cancer. *Cancer Discov* **2024,** *14* (8), 1418-1439.

31. Gu, Y.; Zhang, Z.; Huang, H.; Zhu, W.; Liu, H.; Zhang, R.; Weng, N.; Sun, X., The dual role of CXCL9/SPP1 polarized tumor-associated macrophages in modulating anti-tumor immunity in hepatocellular carcinoma. *Front Immunol* **2025,** *16*, 1528103.

32. Wei, J.; Chen, Z.; Hu, M.; He, Z.; Jiang, D.; Long, J.; Du, H., Characterizing Intercellular Communication of Pan-Cancer Reveals SPP1+ Tumor-Associated Macrophage Expanded in Hypoxia and Promoting Cancer Malignancy Through Single-Cell RNA-Seq Data. *Front Cell Dev Biol* **2021,** *9*, 749210.

33. Li, Y.; Zhao, L.; Li, X. F., Hypoxia and the Tumor Microenvironment. *Technol Cancer Res Treat* **2021,** *20*, 15330338211036304.

34. Sebestyen, A.; Kopper, L.; Danko, T.; Timar, J., Hypoxia Signaling in Cancer: From Basics to Clinical Practice. *Pathol Oncol Res* **2021,** *27*, 1609802.

35. Yan, F.; Teng, Y.; Li, X.; Zhong, Y.; Li, C.; Yan, F.; He, X., Hypoxia promotes non-small cell lung cancer cell stemness, migration, and invasion via promoting glycolysis by lactylation of SOX9. *Cancer Biol Ther* **2024,** *25* (1), 2304161.

36. Korbecki, J.; Kojder, K.; Kapczuk, P.; Kupnicka, P.; Gawronska-Szklarz, B.; Gutowska, I.; Chlubek, D.; Baranowska-Bosiacka, I., The Effect of Hypoxia on the Expression of CXC Chemokines and CXC Chemokine Receptors-A Review of Literature. *Int J Mol Sci* **2021,** *22* (2).

37. Su, Y.; Liu, J.; Tian, Y.; Dong, H.; Shi, M.; Zhang, J.; Li, W.; Huang, Q.; Xiang, N.; Wang, C.; Liu, J.; He, L.; Hu, L.; Haberman, A. M.; Liu, H.; Yang, X., HIF-1alpha Mediates Immunosuppression and Chemoresistance in Colorectal Cancer by Inhibiting CXCL9, -10 and -11. *Biomed Pharmacother* **2024,** *173*, 116427.

38. Enokido, T.; Horie, M.; Yoshino, S.; Suzuki, H. I.; Matsuki, R.; Brunnström, H.; Micke, P.; Nagase, T.; Saito, A.; Miyashita, N., Distinct microRNA Signature and Suppression of ZFP36L1 Define ASCL1-Positive Lung Adenocarcinoma. *Molecular cancer research : MCR* **2024,** *22* (1), 29-40.

39. Planel, S.; Salomon, A.; Jalinot, P.; Feige, J. J.; Cherradi, N., A novel concept in antiangiogenic and antitumoral therapy: multitarget destabilization of short-lived mRNAs by the zinc finger protein ZFP36L1. *Oncogene* **2010,** *29* (45), 5989-6003.

Figures

Figure 1: Cell clustering and annotation of NSCLC cells based on scRNA-seq.

A: Source and types of single cell quantities. **B:** UMAP clustering results of all cells; **C:** Annotation results of all cells. **D:** Marker gene expression for each cell cluster. **E:** Analysis of cell quantities. **F:** Analysis of cell proportions. **G:** Classification UMAP of macrophage subpopulations. **H:** Proportions of macrophage subpopulations. **I:** Annotation results of TAM cells.

Figure 2: Hypoxia decreases the macrophage CXCL9:SPP1 ratio and promotes NSCLC progression.

M0 macrophages induced by PMA using THP-1 cells were cultured under normoxia or hypoxia. **A-B:** qRT-PCR (A) and WB (B) of SPP1 and CXCL9 mRNA and protein levels. **C:** Immunofluorescence staining assessing CXCL9 and SPP1 expression. **D:** Flow cytometric analysis of $CXCL9^{+}/SPP1^{+}$ macrophage ratio. **E:** WB analysis of MMP9 and MMP12 protein levels. **F:** ELISA of IL-7, CXCL10, IL-1β, and CXCL5 levels. M0 macrophages under normoxia or hypoxia were separately co-cultured with A549 or NCI-H2170 cells. **G:** CCK-8 viability assay. **H:** Colony formation assay for cell proliferation. **I:** Flow cytometric analysis of apoptosis. **J:** Scratch migration assay. **K:** Transwell migration and invasion assays. $*p < 0.05$.

Figure 3: ZFP36L1 is a key gene regulating macrophage CXCL9:SPP1 ratio.

A: Correlation analysis between SPP1/CXCL9 and polarization-related transcription regulation factors. **B:** Expression of CITED2 and ZFP36L1 in each TAM subset. **C:** Marker gene expression in each TAM subset. **D:** IHC analysis of ZFP36L1 and SPP1 expression. **E:** Immunofluorescence analysis of ZFP36L1 and SPP1. **F-G:** qRT-PCR (F) and WB (G) of ZFP36L1 in M0 macrophages. ZFP36L1 knockdown M0 macrophages were established and cultured under hypoxia. **H:** WB of

ZFP36L1, SPP1, CXCL9 protein expression. **I:** Immunofluorescence staining assessing CXCL9 and SPP1 expression. **J:** Flow cytometry of CXCL9^{+}/SPP1^{+} ratio in macrophages. **K:** WB of MMP9 and MMP12 protein expression. **L:** ELISA of IL-7, CXCL10, IL-1β, and CXCL5 levels. **M:** ChIP assay validating ZFP36L1 binding to the SPP1 promoter. **N:** Relative luciferase activity in ZFP36L1 overexpressing cells co-transfected with SPP1 wild-type (SPP1-WT) or mutant (SPP1-MUT) promoter constructs. **O:** qRT-PCR detection of SPP1 mRNA expression. ns, not significant; *$p < 0.05$.

Figure 4: ZFP36L1 regulates macrophage CXCL9:SPP1 ratio to influence NSCLC progression in the *in vitro* co-culture system.

M0 macrophages were divided into oe-ZFP36L1+sh-NC, oe-NC+sh-SPP1, oe-ZFP36L1+sh-SPP1, and oe-NC+sh-NC groups. **A-B:** qRT-PCR (A) and WB (B) of ZFP36L1 and SPP1 expression. M0 macrophages from each group were cultured under hypoxia. **C:** WB of MMP9 and MMP12 protein expression. **D:** ELISA of IL-7, CXCL10, IL-1β, CXCL5 levels. Hypoxic M0 macrophages from each group were co-cultured separately with A549 or NCI-H2170 cells. **E:** CCK-8 viability assay. **F:** Colony formation assay for proliferation. **G:** Apoptosis detection by flow cytometry. **H:** Scratch migration assay. **I:** Transwell migration and invasion assays. ns, not significant; *$p < 0.05$.

Figure 5: ZFP36L1 modulates macrophage CXCL9:SPP1 ratio to influence NSCLC progression in a macrophage-organoid co-culture model.

M0 macrophages were divided into oe-ZFP36L1+sh-NC, oe-NC+sh-SPP1, and oe-ZFP36L1+sh-SPP1, and oe-NC+sh-NC groups. M0 macrophages were co-cultured with patient-derived organoids under hypoxia. **A:** H&E staining showing pathological changes in organoids before and after treatment. **B:** EdU staining assessing the proliferation of organoid cells. **C:**

CellTiter-Glo assay for the viability of microtissues. **D-E:** (D) qRT-PCR and (E) WB of apoptotic Caspase 3 and Bcl-2. *$p < 0.05$.

Figure 6: Impact of SPP1⁺ TAMs on the NSCLC microenvironment.

A: GSVA (KEGG) enrichment results of multiple TAM subsets. **B:** Pseudotime analysis of TAM subsets. **C:** Dynamic expression of ZFP36L1, CITED2, SPP1, CXCL9 in TAM subsets during pseudotime. **D:** Cell-cell communication of TAM subsets with other cellular TME components. **E-F:** Communication patterns of $SPP1^+$ TAM as a signal sender (E) or a receiver (F) in a bubble plot.

Figure 7: ZFP36L1-driven $SPP1^+$ TAM accelerates NSCLC progression by binding to CD44 on tumor cells.

A: IHC analysis of SPP1 and CD44 expression. **B:** Immunofluorescence analysis of SPP1 and CD44. CD44 monoclonal antibody was introduced into a co-culture system of M0 macrophages with A549 or NCI-H2170 cells under hypoxic conditions. Experimental groups included: oe-NC+IgG, oe-ZFP36L1+IgG, oe-NC+CD44a, and oe-ZFP36L1+CD44a. **C:** CCK-8 viability assay. **D:** Colony formation assay for proliferation. **E:** Flow cytometry detecting apoptosis. **F:** Scratch migration assay. **G:** Transwell migration and invasion assays. *$p < 0.05$.

Figure 8: ZFP36L1 knockout in macrophage inhibits NSCLC progression by balancing CXCL9:SPP1 ratio *in vivo*.

Mouse models with conditional ZFP36L1 knockout in macrophages were established. **A:** qRT-PCR and WB analyses of ZFP36L1 expression. **B:** Representative images of mouse tumors. **C:** Tumor volume. **D:** Tumor weight. **E:** Histopathological test of tumor tissues by H&E staining. **F:** IHC analysis of ZFP36L1, SPP1, CXCL9, CD44, and Ki67 expression. **G:** Immunofluorescence co-localization of F4/80-positive cells with CXCL9 or SPP1. **H:** ELISA quantification of IL-7,

CXCL10, IL-1β, and CXCL5 levels. *$p < 0.05$.

Sepplementary Fig. 1 Cell clustering and annotation of NSCLC cells based on scRNA-seq.

A: UMI counts (nCount), number of detected genes (nFeature), and mitochondrial gene percentage before quality control. **B:** Point density plots of mitochondrial gene percentage versus nCount (left), nFeature versus nCount (right) before quality control. **C:** nCount, nFeature, and mitochondrial gene percentage after filtering. **D:** Point density plots of mitochondrial gene percentage versus nCount (left), nFeature versus nCount (right) after quality control. **E:** Variation in highly variable gene expression. **F:** Principal component analysis results. **G:** Principal component analysis of macrophages. **H:** UMAP visualization of macrophage subpopulations. **I:** Annotation of macrophage subpopulations.

Sepplementary Fig. 2

A-F: Comparison of the correlation between the ZFP36L1 and CXCL9:SPP1 ratio with the OS and PFI of the patients.

Supplementary Fig. 3 ZFP36L1 regulates macrophage CXCL9:SPP1 ratio to influence NSCLC progression in the *in vitro* co-culture system.

M0 macrophages were divided into oe-NC+IgG, oe-ZFP36L1+IgG, oe-NC+SPP1a, and oe-ZFP36L1+SPP1a groups. **A:** CCK-8 viability assay. **B:** Colony formation for proliferation. **C:** Apoptosis detection by flow cytometry. **D:** Scratch migration assay. **E:** Transwell migration and invasion assays. ns, not significant; *$p < 0.05$.
